# Infection-Free and Enhanced Wound Healing Potential of Alginate Gels Incorporating Silver and Tannylated Calcium Peroxide Nanoparticles

**DOI:** 10.3390/ijms25105196

**Published:** 2024-05-10

**Authors:** Alexandra Catalina Bîrcă, Oana Gherasim, Adelina-Gabriela Niculescu, Alexandru Mihai Grumezescu, Bogdan Ștefan Vasile, Dan Eduard Mihaiescu, Ionela Andreea Neacșu, Ecaterina Andronescu, Roxana Trușcă, Alina Maria Holban, Ariana Hudiță, George-Alexandru Croitoru

**Affiliations:** 1 Department of Science and Engineering of Oxide Materials and Nanomaterials, National University of Science and Technology POLITEHNICA Bucharest, 011061 Bucharest, Romania; ada_birca@yahoo.com (A.C.B.); adelina.niculescu@upb.ro (A.-G.N.); bogdan.vasile@upb.ro (B.Ș.V.); ionela.neacsu@upb.ro (I.A.N.); ecaterina.andronescu@upb.ro (E.A.); truscaroxana@yahoo.com (R.T.); 2 Center for Advanced Research on New Materials, Products and Innovative Processes—CAMPUS Research Institute, National University of Science and Technology POLITEHNICA Bucharest, 060042 Bucharest, Romania; 3 Lasers Department, National Institute for Laser, Plasma and Radiation Physics, 077125 Magurele, Romania; oana.gherasim@inflpr.ro; 4 Research Institute of the University of Bucharest—ICUB, University of Bucharest, 050657 Bucharest, Romania; alina_m_h@yahoo.com (A.M.H.); ariana.hudita@bio.unibuc.ro (A.H.); 5 Department of Organic Chemistry, National University of Science and Technology POLITEHNICA Bucharest, 011061 Bucharest, Romania; danedmih@gmail.com; 6 Department of Microbiology and Immunology, University of Bucharest, 077206 Bucharest, Romania; 7 Department of Biochemistry and Molecular Biology, University of Bucharest, 050095 Bucharest, Romania; 8 Department II, Faculty of Dental Medicine, Carol Davila University of Medicine and Pharmacy, 8 Eroii Sanitari Street, 050474 Bucharest, Romania; alex.croitoru@umfcd.ro

**Keywords:** chronic wounds, silver nanoparticles, tannylated calcium peroxide nanoparticles, wound healing, infection-free, alginate gels

## Abstract

The treatment of chronic wounds involves precise requirements and complex challenges, as the healing process cannot go beyond the inflammatory phase, therefore increasing the healing time and implying a higher risk of opportunistic infection. Following a better understanding of the healing process, oxygen supply has been validated as a therapeutic approach to improve and speed up wound healing. Moreover, the local implications of antimicrobial agents (such as silver-based nano-compounds) significantly support the normal healing process, by combating bacterial contamination and colonization. In this study, silver (S) and tannylated calcium peroxide (CaO_2_@TA) nanoparticles were obtained by adapted microfluidic and precipitation synthesis methods, respectively. After complementary physicochemical evaluation, both types of nanoparticles were loaded in (Alg) alginate-based gels that were further evaluated as possible dressings for wound healing. The obtained composites showed a porous structure and uniform distribution of nanoparticles through the polymeric matrix (evidenced by spectrophotometric analysis and electron microscopy studies), together with a good swelling capacity. The as-proposed gel dressings exhibited a constant and suitable concentration of released oxygen, as shown for up to eight hours (UV–Vis investigation). The biofilm modulation data indicated a synergistic antimicrobial effect between silver and tannylated calcium peroxide nanoparticles, with a prominent inhibitory action against the Gram-positive bacterial biofilm after 48 h. Beneficial effects in the human keratinocytes cultured in contact with the obtained materials were demonstrated by the performed tests, such as MTT, LDH, and NO.

## 1. Introduction

Treating skin wounds is a serious clinical problem that requires optimal and improved solutions to overcome all possible situations that arise unconditionally. When it comes to the healing process, the wound type is an important aspect that must be considered, because it is well known that superficial tissue injuries might be healed more rapidly than critical chronic wounds.

In the case of severe chronic wounds, the healing process is stopped at the inflammatory phase, where the growth factors and extracellular matrix (which are fundamental for healing) are influenced in a harmful way by the increased levels of inflammatory mediators. Consequently, a longer healing time leads to increased microbial susceptibility and infection risk at the wound site. Infections, in turn, have a negative effect on maintaining optimal levels of growth factors and oxygen supply, thus altering the activation and support steps of the healing process in affected tissues. High levels of bacteria often become complex and self-organized structures (biofilms), in which development is promoted by the environment of chronic wounds [1,2,3,4,5]. Besides opportunistic contamination and colonization, hypoxia is an outcome of chronic wounds that hampers the fibroblast proliferation and collagen generation. During inflammation, the oxygen supply is combinedly limited by the imbalance of growth factors and the impaired healing process [6]. However, the healing process is delayed and hindered when the oxygen level at the wound site is low for a longer period. As a result, the infection risk increases, so there is a pressing need to develop new and effective ways to treat chronic wounds [7].

Current strategies in wound care management and treatment involve the topical use of dressings made of various materials with specific properties, depending on the necessity of affected tissues. Therefore, the process of designing, developing, and validating novel composites as wound dressings is still a demand to achieve improved and infection-free healing. The wound dressing candidate should fulfill all the required features for an ideal dressing or at least close to the ideal one [8,9,10].

One essential characteristic of a wound dressing is its moisture retention, which is important in maintaining a proper environment for the healing process. George Winter conceptualized this parameter, thus making a revolutionary transition from classical dry dressings to moist materials that more efficiently support the wound healing process by helping the affected tissue from the outside. According to these moisture-promoting properties, many types of dressings have been developed, including hydrogels, hydrocolloids, films, and foams [11,12,13]. Among these, hydrogels represent effective wound healing candidates, as they are easy to produce and optimize and have an impressive and tunable capacity for incorporating certain molecules or materials, thus resulting in dressings with synergistic therapeutic activity that effectively reach several parameters necessary for normal tissue healing. It is important to keep the appropriate conditions of the wound/dressing microclimate by providing a wet and cooling setting, as well as a soothing effect that helps reduce the pain [14,15,16,17].

Considering the required features of a wound dressing, the selection of a proper material is a very important criterion when developing solutions for wound treatment. In this sense, the decision between natural or synthetic polymers considers their interactions with the affected tissues. The use of natural polymers is preferred for topical applications, given their economic implications, together with intrinsic biocompatibility, biodegradability, derivatization, and functionalization versatility, which lead to favorable activity when in contact with the human body and the environment. However, some limitations occur when using natural polymers, mostly due to their low mechanical properties. Such outcomes may be overcome by including a synthetic polymer, increasing the reticulation degree or by modifying the natural polymer. For example, chitosan exhibits increased swelling and degradation properties when it is modified with organic compounds (trans-aconitic acid or trimellitic anhydride) or inorganic additives (aluminum chloride, aluminum sulfate, or iron sulfate) [18,19]. Polysaccharides such as chitosan, cellulose, starch, alginate, and hyaluronic acid are natural polymers that have been extensively investigated and confirmed to fabricate effective wound dressings [1,20,21,22].

Among the many beneficial characteristics for biomedical use, sodium alginate (Alg) possesses two necessary properties that recommend it for developing hydrogels, namely biocompatibility and biodegradability. In the case of this natural polysaccharide, instant gelation can easily be achieved through contact with multivalent cations, the most used being Ca^2+^ [23,24]. Owing to the natural characteristic of alginate (a copolymer of guluronic acid and mannuronic acid), and easy conditions of use and processing, Alg-based biomaterials have been intensively studied for modern biomedicine, especially during the last decades [25,26].

It is worth mentioning that hydrogels have the capacity to be formulated as to exhibit a specific controlled release of a drug or active biomolecule, including antimicrobial agents, thus being included in the category of interactive dressings [11,15].

Developing a therapeutic dressing biomaterial that could control or intercept opportunistic events in skin wounds is highly demanded. Once attached to the injured tissue (contamination), opportunistic bacteria can undergo colonization and further biofilm development very quickly and harmfully. More than causing a moderate-to-severe systemic immune response, the healing process of biofilm-complicated wounds is delayed. In such case, the use of conventional antimicrobial drugs is less effective, as they do not have the ability to withstand the biofilm-embedded microorganisms because of their intricate and very strong resistance mechanisms [27,28].

Considering the biomedical implications of nanotechnology, the use of nanoparticles in wound dressing formulations, with a focus on their bactericidal or biostatic effects, is a valuable and dynamic choice to struggle with the highly disturbing possibility of wound infection. Owing to their diversified and validated anti-pathogenic action, silver nanoparticles still stand up as a considerable approach to limit or eradicate clinically relevant microbial biofilms, exerting broad-spectrum activity and without exhibiting bacterial resistance [29,30,31,32,33,34]. The biological properties of silver nanoparticles are strongly influenced by their morphology, size, and crystallinity, which can be nowadays controlled by using a microfluidic synthesis process. Small reactant volumes flow through a microfluidic platform and the silver reduction reaction occurs within micro-reactors [35], resulting in the formation of nanoparticles with particular morphology, smaller particle size, and homogenous particle size distribution [36,37,38]. Moreover, the process and product reproducibility is an indisputable advantage of the microfluidic synthesis of silver nanoparticles. Safe and improved manipulation of reagents, small solution volumes, controllable reaction conditions, optimization possibilities (by adjusting different parameters, such as the platform design, tubing diameter, rotational speed, local temperature, type and concentration of reagents), size-related increased reactivity, and facile collecting of the resulting nanoparticles, are some of the advantages of using a microfluidic platform for synthesizing silver nanoparticles [39,40,41,42].

To overcome the hypoxia issue in chronic wounds, a variety of materials, such as hydrogen peroxide, magnesium peroxide, calcium peroxide, and sodium percarbonate, have been evaluated for their capacity to produce hydrogen peroxide (H_2_O_2_) following a reaction with water, which further undergo a decomposition reaction that generates oxygen. Among these materials, calcium peroxide (CaO_2_) nanoparticles are biocompatible and biodegradable, and possess an attractive capacity to generate oxygen, being suitable for specific biomedical applications. Depending on the pH conditions, the release of oxygen happens, especially in an acidic environment (as in the case of hypoxia-mediated status of chronic wounds). CaO_2_ has been confirmed as a safe and effective material in biomedical applications, but greater attention is needed when it comes to the production of hydrogen peroxide. In this sense, embedding CaO_2_ nanoparticles in alginate hydrogels is an attractive strategy to support oxygen releasing at the wound level, providing a proper method to fulfill wound healing requirements. When applied to a chronic wound, Alg hydrogels decorated with CaO_2_ nanoparticles will gradually generate oxygen, and the Ca^2+^ originating from the CaO_2_–water reaction will beneficially support local cellular events, promoting cell proliferation and stimulating the regeneration process [43,44,45,46,47,48].

Tannic acid (TA), a biocompatible and biodegradable substance, is extracted from various natural sources and part of the polyphenols class. Given that TA is easy to find and obtain, it may easily interact with polymers and exhibit intrinsic therapeutic effects (antimicrobial, anti-inflammatory, antioxidant, and anticancer effects), tannylated nanomaterials have been successfully investigated for biomedical applications. This natural polyphenol can be used in composite hydrogels as a stabilizing agent in CaO_2_ synthesis, a reducing agent in silver nanoparticle synthesis, as a cross-linking agent for certain hydrogels, and so on [49,50,51,52].

Our study presents an innovative dressing solution for the management of chronic wounds. Tannylated calcium peroxide (CaO_2_@TA) and silver (S) nanoparticles embedded in alginate-based hydrogels are proposed to improve oxygenation and inhibit opportunistic infection, respectively, synergistically contributing to an improved healing capacity.

## 2. Results

### 2.1. Characterization and Optimization of Silver Nanoparticles

**Figure 1:**
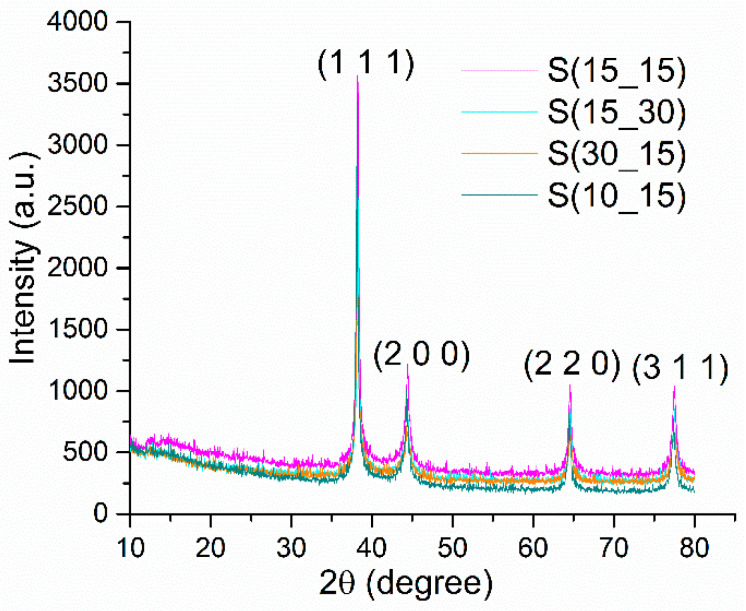
X-ray diffraction (XRD) patterns for S(15_15), S(15_30), S(30_15), S(10_15) silver samples.

The X-ray diffraction (XRD) patterns of silver samples synthesized by the microfluidic method are shown in Figure 1.

The phase identification was made by indexing the strong Bragg reflections in the diffraction patterns and then assigning them to Miller indices. For all samples, the only identified phase was silver in a cubic crystal system with Fm-3 m space group symmetry, according to the 04-014-0266 reference code from the PDF-ICDD database. The representative (1 1 1), (2 0 0), (2 2 0), and (3 1 1) diffraction planes for face-centered silver crystals were observed in recorded diffractograms. By considering the intensity variation corresponding to the (1 1 1) reflection in all samples, the S(15_15) silver specimen had the best outcome in terms of crystallinity degree. However, all silver samples presented a good crystallinity, with sharp and broad peaks, suggesting the small sizes of crystallites.

The average crystallite size was calculated using the Debye–Scherrer formula:

(1) $$D=\frac{0.9\lambda }{\beta cos\theta }$$

where D is the crystallite size, determined by considering the following parameters: λ represents the wavelength of the X-ray (0.1540 nm, in our case), β is known as the full width at half maximum (FWHM), and θ represents the diffraction angle.

Table 1 outlines the crystallite size values obtained for the S(15_15), S(15_30), S(30_15), and S(10_15) samples. The smallest crystallite size was found for the S(30_15) sample, where the input speed for silver nitrate solution was set to 30 RPM, and a 15 RPM speed was chosen for each of the other two channels. Samples S(15_15) and S(15_30) had similar results for crystallite size calculations, and S(10_15) showed the highest crystallite value, with only a ~0.4 nm variation. By considering these results and for a better understanding of the implications of the microfluidic synthesis on the formation of silver nanoparticles, we observed a decrease in the crystallite size with a decrease in the input of the reducing agent. The smallest crystallite size was achieved for the S(15_30) sample, while the biggest crystallite size was found in the S(10_15) sample, corresponding to 1:1 and 1:3 ratio between the admission speeds (RPM) for silver nitrate–ascorbic acid, respectively.

**Figure 2:**
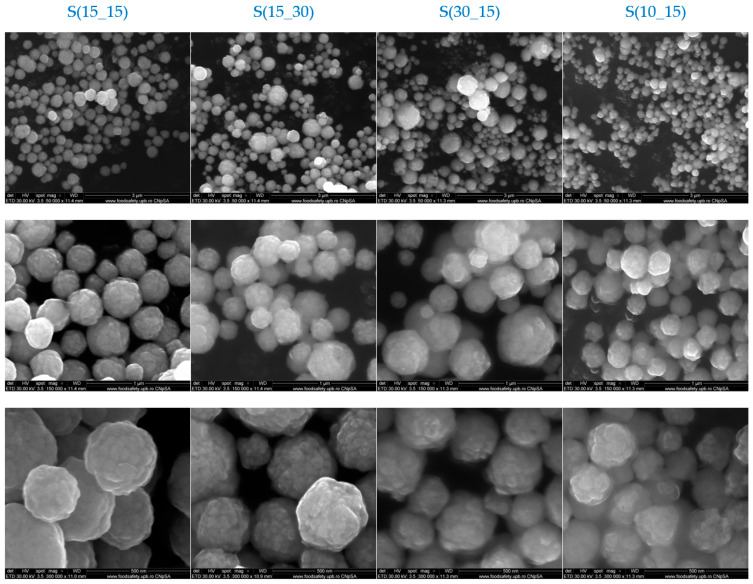

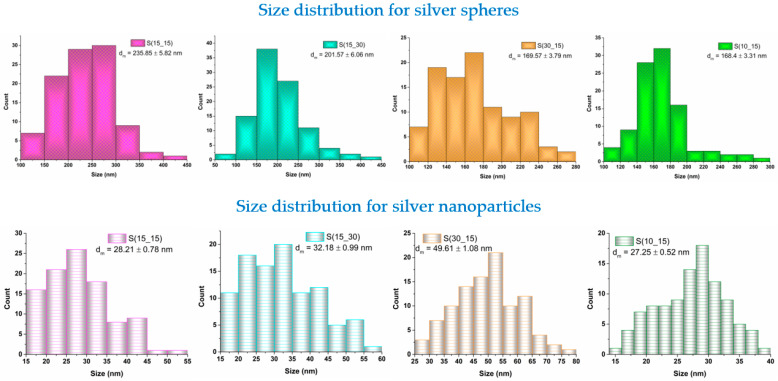
Scanning electron microscopy (SEM) micrographs at different magnifications and size distribution histograms (measurements made on silver spheres, first set of histograms, and constituent silver nanoparticles, second set of histograms) for silver samples.

Scanning electron microscopy (SEM) analysis was used to evaluate the morphology and size of the obtained silver samples, as evidenced through the micrographs and graphical representations from Figure 2.

The SEM micrographs evidenced a particular arrangement of the silver nanoparticles in the form of micron-sized silver spheres. The reducing agent generally significantly affects the morphology of synthesized silver particles. In our case, the utilization of ascorbic acid determined the formation of individual silver nanoparticles, which aggregated in the form of spheres. Depending on the parameters during the microfluidic synthesis, some differences between the four silver samples were noticed in terms of morphology and size uniformity of the spheres. The micrographs pointed out that the most accurate sphere shape was attained for the S(15_15) sample, where the input speed for the metallic solution and both reducing solutions was set to 15 RPM. The S(15_30) sample, obtained with double speeded reducing solutions, also presented a quasi-spherical morphology, but in the case of the S(30_15) and S(10_15) samples, some conformational defects that did not allow for maintaining a sphere-type attribution were noticed. This effect, associated with the admission speed of reactants during the microfluidic synthesis, was correlated with the reaction process stages, namely either an accelerated nucleation and incomplete maturation of particles (S(30_15)), or a complete maturation of insufficiently nucleated particle cores (S(15_30) and S(10_15)). The size distribution histograms were obtained by measuring the size of spheres and constituent nanoparticles. In terms of sphere dimensions, the S(15_15) sample displayed the biggest value, while much smaller and similar sphere sizes were observed for the S(10_15) and S(30_15) samples. In terms of nanoparticle dimensions, maximal and minimal values corresponded to the S(30_15) and S(10_15) samples, respectively. These observations considered the calculated average sizes of particles, but represented histograms showed a particular homogeneity in the size of S(15_15) silver spheres and nanoparticles. This observation is important when silver is synthetized through a controlled microfluidic platform, especially in the realm of biomedicine, because the spherical shape and the dimensional uniformity are critical characteristics for proper results.

**Figure 3:**
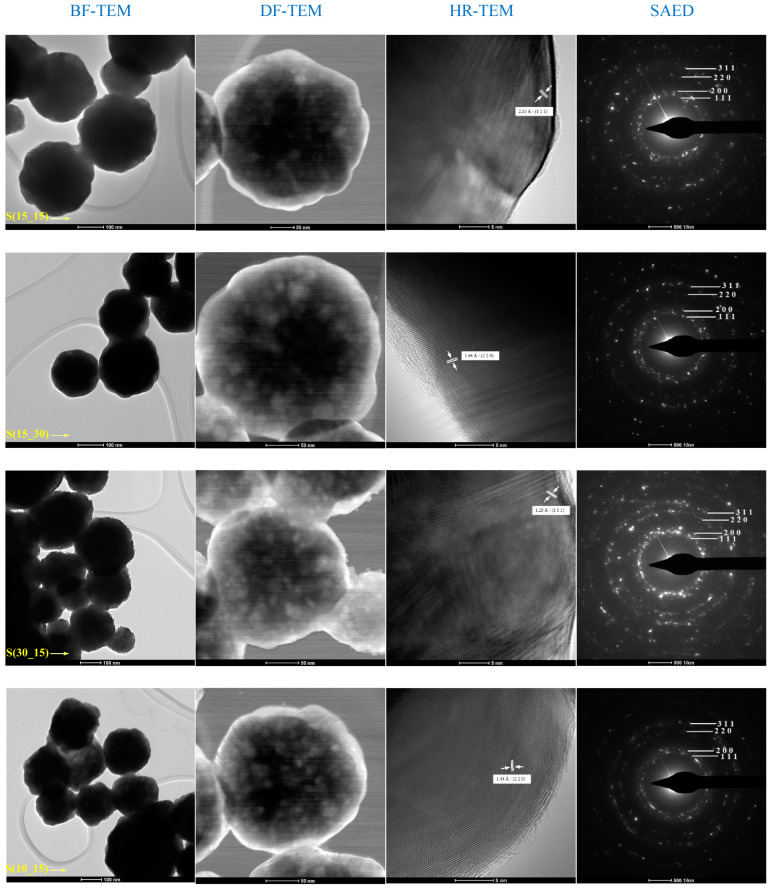
Bright-field (BF), dark-field (DF), and high-resolution (HR) transmission electron microscopy (TEM) micrographs for S(15_15), S(15_30), S(30_15), and S(10_15) silver samples, with corresponding selected area electron diffraction (SAED) patterns.

Transmission electron microscopy (TEM) analysis was used for the in-depth observation of the morphological and microstructural features of the silver samples. The collected data for S(15_15), S(15_30), S(30_15), and S(10_15) samples are displayed in Figure 3.

TEM micrographs were performed in the dark-field (DF), bright-field (BF), and high-resolution (HR) modes for all silver powdery samples (S(15_15), S(15_30), S(30_15), and S(10_15)). DF-TEM provides images of scattered electrons, while BF-TEM gives images of transmitted electrons. In the bright-field mode, the objective aperture increases the contrast by blocking the scattered electrons; therefore, crystalline materials are recorded as darker. In the dark-field mode, a tilted incident electron beam and obstructed transmitted electrons provide enhanced contrast for the accurate visualization of ultra-small structures [53,54], like the silver nanoparticles that form the silver spheres in our study. The TEM micrographs revealed that the silver spheres consisted of silver nanoparticle agglomerations. Regarding the morphology of the spheres, TEM results were compliant with SEM observations, as the S(15_15) sample possessed a well-defined spherical shape, while the S(15_30) sample had a quasi-spherical aspect. In the case of S(30_15) silver, free and ultra-small nanoparticles were noticed on the circle’s edge that limited the main sphere. Also, the S(10_15) sample consisted of mixed quasi-spherical and polyhedral silver spheres. The HR-TEM images and SAED patterns confirmed the Fm-3 m cubic structure for all silver samples, evidencing the presence of (1 1 1), (2 0 0), (2 2 0), and (311) lattice planes corroborated with measured interplanar distances.

**Figure 4:**
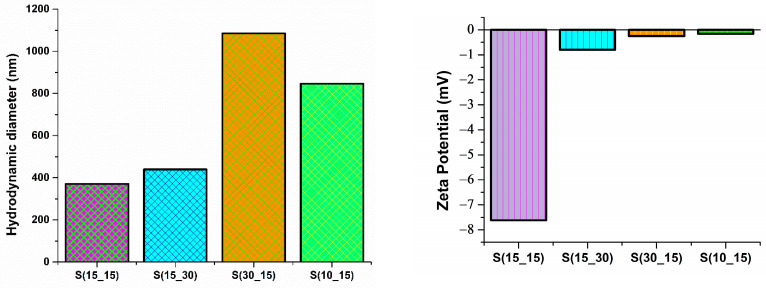
Dynamic light scattering (DLS) results as hydrodynamic diameter (nm), left, and zeta potential (mV), right, for S(15_15), S(15_30), S(30_15), and S(10_15) silver samples.

The graphic representation of the hydrodynamic diameter (nm) is observed in Figure 4. The surface charge is expressed as zeta potential (mV) and the values are measured by DLS for S(15_15), S(15_30), S(30_15), and S(10_15) silver samples.

In the hydrodynamic diameter (nm) graph, the differences noticed between the analyzed samples provided relevant information for the identification of the most suitable silver sample for further biomedical applications. The hydrodynamic diameter measured for the S(15_15) silver sample was 370 nm, which was the smallest value, in contrast with the S(30_15) sample (1084 nm). The S(15_30) and S(10_15) silver samples had diameters of 439 nm and 846 nm, respectively. In compliance with SEM observations and size distribution histograms (Figure 2), the DLS results evidenced smaller hydrodynamic sizes for S(15_15) and S(15_30). In the case of the S(30_15) and S(10_15) samples, the much bigger hydrodynamic diameter was related to the defective silver spheres, which are not stable and tend to form large agglomerates. This explanation also considered the results given by the zeta potential measurements. We can see that all samples had a negative charge, which is a beneficial feature for materials used in biomedical applications. However, the stability of silver spheres cannot be included with certainty because the zeta potential values did not exceed the maximal value of −7.62 mV recorded for the S(15_15) sample. The surface charge for the other silver samples (S(15_30), S(30_15), and S(10_15)) was around −0.40 on average. Considering the hydrodynamic diameter and zeta potential results, but also previous microstructural and morphological observations, we concluded that the S(15_15) sample exhibited the required characteristics for biomedical applications, representing the optimized silver-based candidate for the development of enhanced wound healing hydrogels.

### 2.2. Characterization and Evaluation of Tannylated Calcium Peroxide Nanoparticles

**Figure 5:**
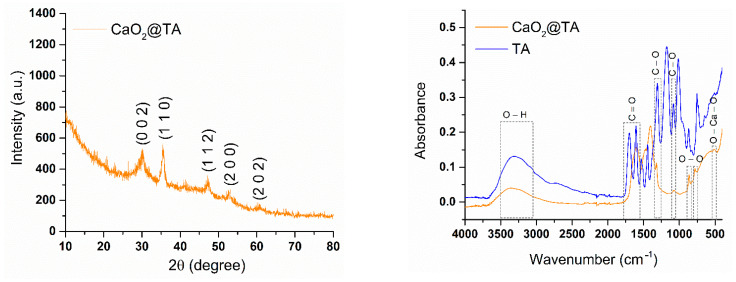
X-ray diffraction (XRD) pattern (**left**) and Fourier transform infrared spectroscopy (FTIR) spectra (**right**) recorded for CaO_2_@TA.

X-ray diffraction analysis was used to obtain relevant crystallographic and microstructural information on the CaO_2_@TA powder, while FTIR analysis was comparatively performed on the CaO_2_@TA sample and pure TA substance for compositional investigation. The collected diffractogram and spectral data are presented in Figure 5.

Diffraction planes with Miller indices of (0 0 2), (1 1 0), (1 1 2), (2 0 0), and (2 0 2) were identified in the XRD results, according to the 00-003-0865 reference code for calcium peroxide as a single phase, based on the PDF-ICDD database. The CaO_2_@TA sample had a tetragonal structure, with an I4/mmm space group and moderate crystallinity due to the increased amount of TA organic phase. However, the crystallite size for the CaO_2_@TA powder (determined with the same Debye–Scherrer formula) was estimated to be 47.34 nm. The functional group identification within the FTIR results confirmed the successful formation of tannylated calcium peroxide nanoparticles by the selected precipitation protocol. The O-H functional group appeared in both spectra at 3050–3500 cm^−1^ (with a maximum absorption at ~3364 cm^−1^), suggesting the samples’ hydrophilicity. The fingerprint of CaO_2_ was only observed for the CaO_2_@TA sample by the presence of an O-Ca-O vibrational band at ~517 cm^−1^. Two stretching modes, at 780 and 863 cm^−1^ wavenumbers, were indicative of the O-O functional group of peroxide ions. TA-originating vibrations were specifically identified at ~1700 and ~1595 cm^−1^ (conjugated C=O stretching), ~1480 cm^−1^ (aromatic C=C stretching), and ~750 cm^−1^ (C=C deformation) [55,56].

**Figure 6:**
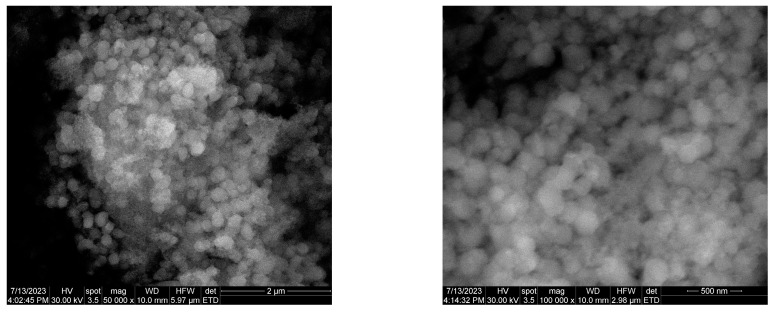

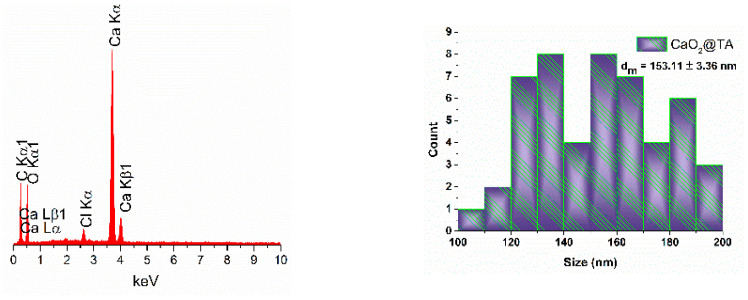
Scanning electron microscopy (SEM) micrographs at different magnifications, energy-dispersive spectroscopy (EDS) spectrum, and size distribution histogram for CaO_2_@TA nanoparticles.

The morphological, dimensional, and elemental investigation was obtained through SEM, together with the size distribution of particles and EDS analysis. The results are presented in Figure 6.

The CaO_2_@TA sample consisted of individual nanosized, spherical particles with a tendency of agglomeration. EDS results pointed out specific elements from the sample, such as Ca and O, which are characteristic for calcium peroxide. In terms of particle size, the SEM micrographs revealed the formation of particles with an increased dimensional uniformity. This fact was also observed in the size distribution histogram, which also provided the average dimension of the CaO_2_@TA particles (153.11 ± 3.36 nm).

**Figure 7:**
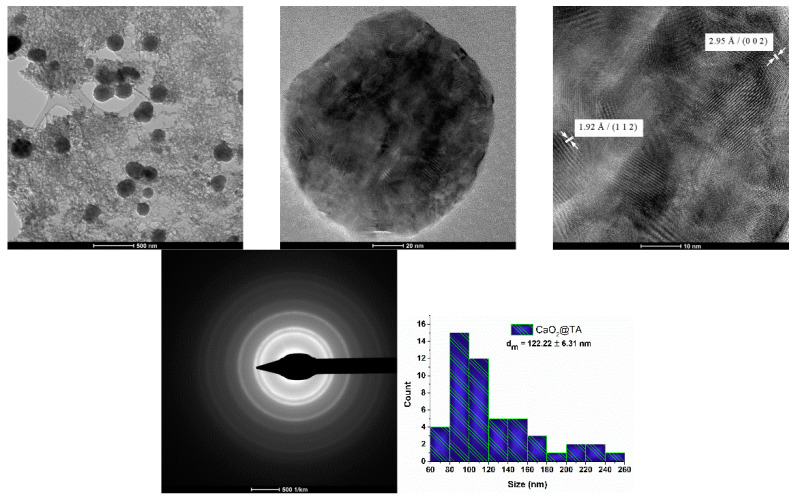
Bright-field (BF) and high-resolution (HR) transmission electron microscopy (TEM) micrographs (first row), and corresponding selected area electron diffraction (SAED) pattern, and size distribution histogram (second row) for CaO_2_@TA nanoparticles.

For a better observation of the shape and size of synthesized CaO_2_@TA nanoparticles, TEM analysis was performed. The TEM results, including bright-field and high-resolution micrographs, together with the SAED pattern and size distribution histogram, are presented in Figure 7.

TEM analysis demonstrated the presence of two phases, namely the CaO_2_ nanoparticles (dark areas) and the TA matrix (observed as a homogenous “lace” model that shielded the CaO_2_ nanoparticles). The spherical shape and a detailed polycrystalline structure of the particles were noticed. The distance between the planes of atoms, measured from the HR-TEM data, confirmed different crystallite growth directions in accordance with the XRD results. The SAED pattern pointed out the polycrystalline structure of the sample, the reduced crystallinity, and the tetragonal crystal structure of CaO_2_ (specific luminous rings corresponding to previously identified lattice planes). Size distribution has also been obtained for CaO_2_@TA nanoparticles, revealing an average calculated dimension of 122.22 ± 6.31 nm. However, the size of most nanoparticles was predominantly outlined between 80 and 100 nm.

**Figure 8:**
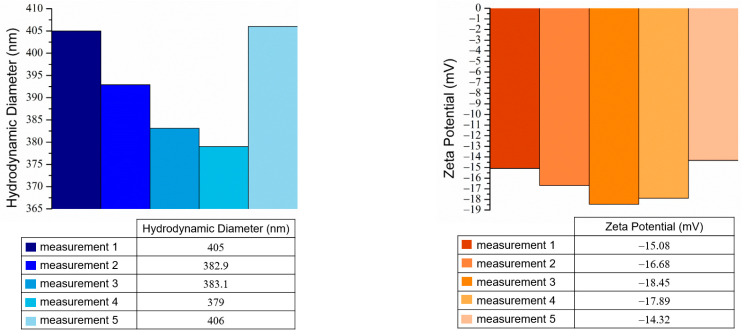
Dynamic light scattering (DLS) results as hydrodynamic diameter (nm), left, and zeta potential (mV), right, for CaO_2_@TA nanoparticles.

The spherical morphology of the CaO_2_@TA nanoparticles lent them the suitability for performing the DLS analysis, which determined the hydrodynamic diameter (nm), as well as the zeta potential (mV) associated with the surface charge of the nanoparticles. Figure 8 shows the as-measured parameters for CaO_2_@TA nanoparticles, the mean values being estimated after five individual measurements.

As expected, the hydrodynamic dimensional values were substantially increased compared to the average particle sizes determined from the electron microscopy analyses. The mean hydrodynamic diameter was estimated to be 393.2 nm for the CaO_2_@TA sample, while zeta potential measurements revealed the formation of nanoparticles with a negative surface charge, which is a favorable aspect for nanoparticles used in biomedical applications. The mean value of 򲈒16.48 mV indicated an increased stability of the CaO_2_@TA nanoparticles.

**Figure 9:**
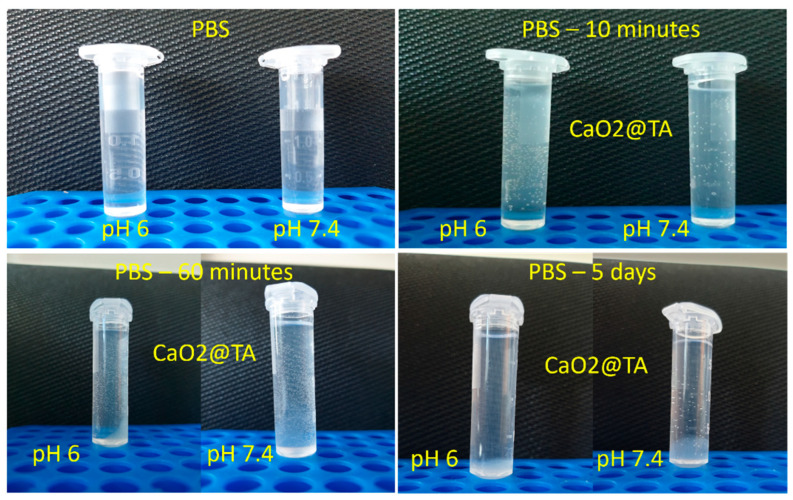
Oxygen bubble visualization for CaO_2_@TA nanoparticles in PBS solution at pH 6 and pH 7.4 at different time points, revealing their oxygen-releasing ability.

Oxygen bubble visualization was possible using a phone camera by taking pictures of the CaO_2_@TA sample in PBS solutions at different time points (Figure 9). Some studies on calcium peroxide nanoparticles reported an increased release of oxygen under acidic conditions. Therefore, we tested the ability of tannylated nanoparticles for oxygen bubble formation after immersion in PBS with different pH values, namely pH 6 and pH 7.4.

A considerable difference between the ability of our sample to generate oxygen bubbles at pH 6 and pH 7.4 was observed after only 10 min of reaction. Though dimensionally reduced, many more bubbles were generated under acidic conditions compared to neutral conditions. Bubbles with similar sizes were observed after 60 min, regardless of the testing media, but their amount was slightly increased for the CaO_2_@TA sample tested at a pH of 7.4. After 5 days of testing, no bubbles were identified under acidic conditions, while few oxygen bubbles were still noticed in the neutral solution.

### 2.3. Investigation of Optimized Alginate-Based Hydrogel Formulations

**Figure 10:**
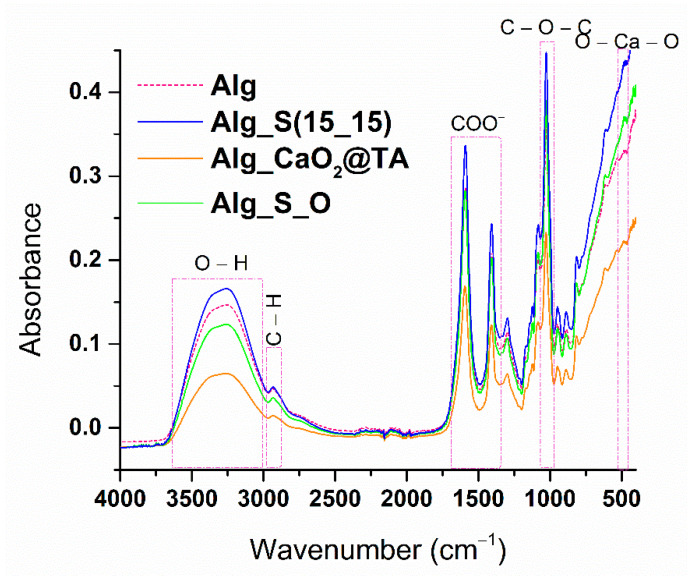
Fourier transform infrared spectroscopy (FTIR) spectra recorded for Alg, Alg_S(15_15), Alg_CaO_2_@TA, and Alg_S_O samples.

After the synthesis and physicochemical characterization of silver and calcium peroxide nanoparticles, the next step was to embed them within an alginate-based hydrogel formulation, with the aim of developing an innovative wound dressing with antimicrobial and oxygen-releasing properties. Among the four silver samples obtained using the microfluidic approach, the S(15_15) sample was identified as the optimal candidate for developing improved hydrogels for such specific applications. Moreover, the synthesized CaO_2_@TA nanoparticles exhibited suitable properties to be incorporated in hydrogels with a role in oxygen release. Sequential investigations on four hydrogel formulations, namely alginate (Alg), silver-modified alginate (Alg_S(15_15)), calcium peroxide-modified alginate (Alg_CaO_2_@TA), and alginate modified with silver and tannylated calcium peroxide nanoparticles (Alg_S_O), were performed. All hydrogels were subjected to FTIR analysis, and the collected spectra are included in Figure 10.

Since alginate was the basic polymeric matrix of the herein-developed wound dressing materials, FTIR spectra presented similar vibrational bands for all samples. Their hydrophilic character was revealed by the typical large band of hydroxyls, with a maximum absorption at about 3200 cm^−1^. A decreased absorbance was observed for samples loaded with CaO_2_@TA nanoparticles (namely, Alg_CaO_2_@TA and Alg_S_O hydrogels) in the 3050–3500 cm^−1^ range due to the interactions between the polymer matrix and calcium ions, which have an active role in the cross-linking of sodium alginate. Alginate-originating vibrations were specifically identified at 2934, 1593, 1409, and 1027 cm^−1^, corresponding to C–H symmetric stretching, COO^−^ asymmetric and symmetric vibrations, and C–O–C asymmetric stretching, respectively. Collectively, the FTIR results demonstrated the successful formation of alginate-based composite hydrogels, modified either with silver or tannylated calcium peroxide nanoparticles or with both nanoparticles, as prospective wound dressings.

**Figure 11:**
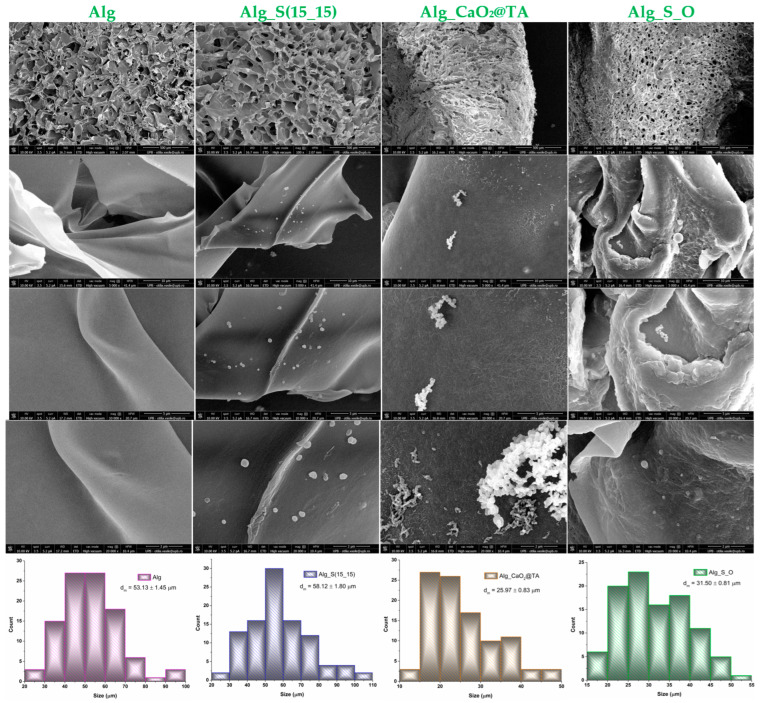
Scanning electron microscopy (SEM) micrographs for Alg, Alg_S(15_15), Alg_CaO_2_TA, and Alg_S_O hydrogels at different magnifications, with corresponding pore size distribution.

SEM analysis was performed on all four hydrogels to observe their microstructure (including porosity) and distribution of S(15_15) and CaO_2_@TA nanoparticles through the alginate matrix. The micrographs were captured at different magnifications (100×, 5000×, 10,000×, and 20,000×) and are included in Figure 11, together with the corresponding pore size histograms.

In the overall SEM images at low magnification (100x), it was easy to see and attribute the pore-holding property for all four obtained hydrogels. Some evident differences in the size and shape of the pores between CaO_2_@TA-loaded (Alg_CaO_2_@TA and Alg_S_O) and CaO_2_@TA-free (Alg and Alg_S(15_15)) hydrogels were noticed, due to the reticulation of alginate matrix by calcium ions. This observation was supported by the measurement and size distribution of the pores. Similar pore dimensions were characteristic for the Alg and Alg_S(15_15) samples, with an average size of 53.13 ± 1.45 µm and 58.12 ± 1.80 µm, respectively. Loading CaO_2_@TA nanoparticles led to a decrease in the size of hydrogel pores, with average pore dimensions of 25.97 ± 0.83 µm and 31.50 ± 0.81 µm for the Alg_CaO_2_@TA and Alg_S_O samples, respectively, which confirmed the ionic cross-linking of the alginate matrix. The presence of CaO_2_@TA nanoparticles also influenced the porosity ratio and pore distribution within the hydrogels subjected to lyophilization, representing an advantage for obtaining dressings with homogeneous porous structure. SEM micrographs at higher magnifications showed that individual particles of S(15_15) and CaO_2_@TA preserved their morphology and dimension after embedding in the alginate matrix. Still, some differences have been noticed in terms of distribution. The Alg_S(15_15) sample contained S(15_15) spheres that were homogeneously and individually distributed within the polymer matrix, while the Alg_CaO_2_@TA sample exhibited a homogeneous distribution of aggregated CaO_2_@TA nanoparticles. Such fibrous aggregation of CaO_2_@TA nanoparticles in contact with the alginate might have resulted from the Ca^2+^-mediated interactions between the tannylated particles and the polysaccharide matrix.

**Figure 12:**
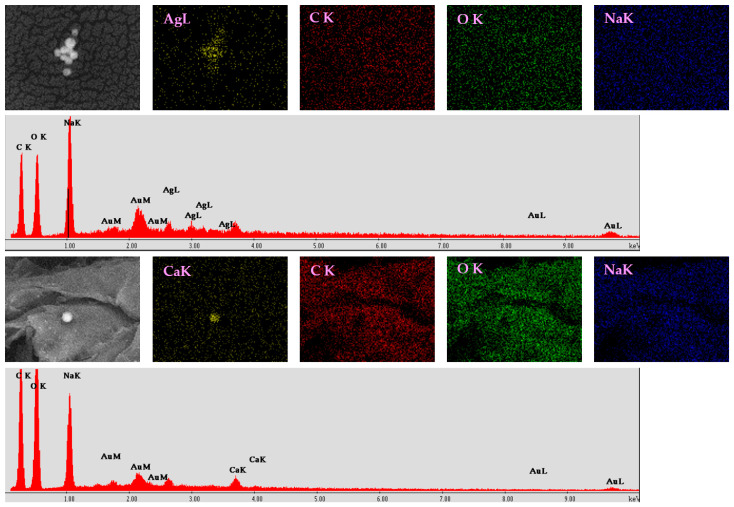
Scanning electron microscopy (SEM) micrographs with corresponding elemental mapping records (rows 1 and 3) and energy-dispersive spectroscopy (EDS) spectra (rows 2 and 4) recorded for Alg_S(15_15) (rows 1 and 2) and Alg_CaO_2_@TA (rows 3 and 4) hydrogels.

Because of the similarities between the morphology and size of silver and tannylated calcium peroxide nanoparticles distributed within the alginate matrix, hydrogels were further investigated using EDS analysis. Besides EDS spectra (revealing elemental composition), mapping representations were collected for Alg_CaO_2_@TA and Alg_S(15_15) samples (Figure 12).

The results obtained for Alg_S(15_15) hydrogel revealed the presence of silver–AgL together with carbon, oxygen, and sodium originating from the alginate polymeric matrix.

The elemental mapping representation of Alg_CaO_2_@TA hydrogel indicated the presence of calcium, CaK, along with the EDS spectrum where the appearance of the Alg-originating characteristic elements (carbon, oxygen, and sodium) has also been identified.

**Figure 13:**
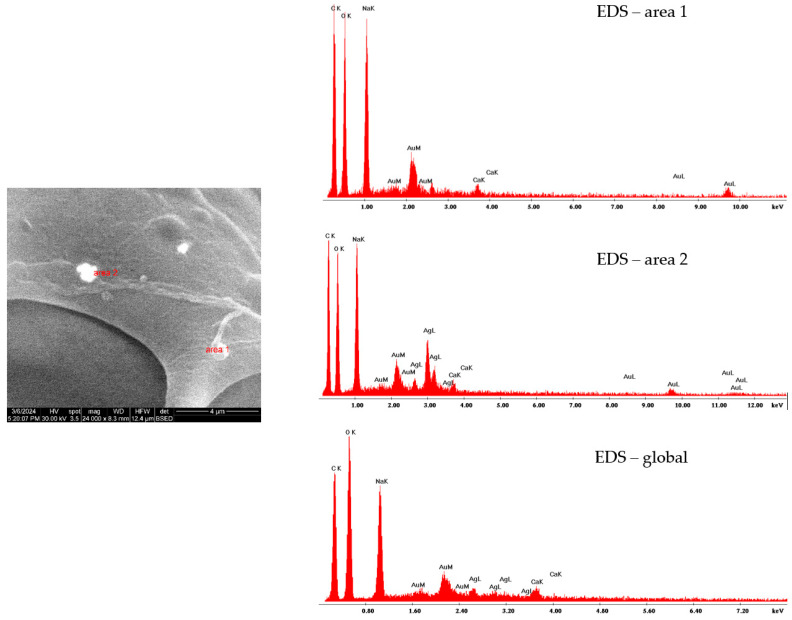
Scanning electron microscopy (SEM) micrograph and corresponding energy-dispersive spectroscopy (EDS) spectra recorded for Alg_S_O hydrogel.

To evaluate the presence of either silver or calcium peroxide in the alginate matrix for the Alg_S_O hydrogel, EDS analyses were performed on two distinct areas (SEM micrographs were collected in backscatter mode, the silver (area 2) being easier to spot compared to calcium peroxide (area 1) due to the increased brightness induced by the heavier element), as well as globally (Figure 13). The EDS spectrum for area 1 highlighted the presence of calcium, while the spectral EDS data for area 2 revealed the presence of silver and calcium as well because of the interaction between alginate and calcium ions. However, the global EDS spectrum of Alg_S_O hydrogel proved the concomitant presence of silver and calcium elements, thus confirming that both types of nanoparticles were successfully embedded and distributed in the alginate matrix.

**Figure 14:**
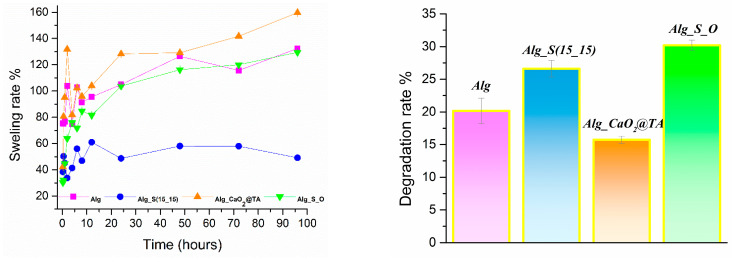
Graphical representation of the swelling rate for Alg, Alg_S(15_15), Alg_CaO_2_@TA, and Alg_S_O hydrogels (15 min–96 hours’ time interval), left, and the corresponding degradation rate (after 2 weeks), right. All results represent the mean values of three independent experiments ± standard deviation (S.D.).

The swelling rate is an essential parameter that must be evaluated when assessing if a hydrogel-type dressing meets the critical conditions for wound healing. When in contact with a biologically simulated medium, like the herein selected SBF, the behavior of hydrogels shows quite a large variation in the first minutes of interaction, and later, they stabilize and maintain a constant absorption rate. To observe the initial behavior of alginate-based hydrogels, the synthesized samples were immersed in SBF solution and evaluated, in terms of mass variation, after 15, 30, and 60 min. Further measurements were performed every 2 h until 12 h, then once a day until 96 h. After 4 days of bio-simulated testing, all samples were evaluated from the degradation point of view. The recorded results for Alg, Alg_S(15_15), Alg_CaO_2_@TA, and Alg_S_O hydrogels are displayed in Figure 14.

At first look, we noticed that the Alg_S(15_15) hydrogel exhibited the lowest degree of swelling compared to all other samples. In contrast, the highest swelling rate (159%) was achieved for the Alg_CaO_2_@TA hydrogel. Though slightly reduced, a similar swelling behavior was noticed for Alg_S_O and Alg hydrogels after 96 h, with maximal values of 129% and 132%, respectively. These results indicated that, even after 96 h, the proposed dressing materials exhibited a good swelling capacity, which represents a favorable requirement for proper wound healing. The degradation results indicated a total degradation rate below ~30% after 2 weeks of evaluation in SBF, regardless of the hydrogel composition. The least degraded material was the Alg_CaO_2_@TA hydrogel, with a 15% degradation rate, while the highest rate of degradation was observed for the Alg_S_O sample (30%). The Alg_S(15_15) hydrogel recorded a degradation rate of 26%, which was the second in line after the three-component hydrogel.

**Figure 15:**
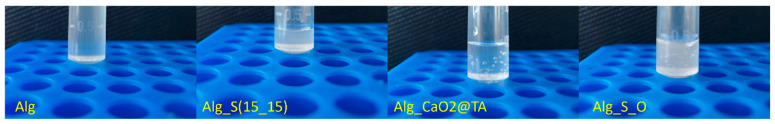
Oxygen bubble visualization for Alg, Alg_S(15_15), Alg_CaO_2_@TA, and Alg_S_O hydrogels stored for 7 days at room temperature.

Oxygen-releasing hydrogels even show this property in a naked-eye manner by observing the formation of gas bubbles with time. In Figure 15, the bubble formation was only observed for Alg_CaO_2_@TA and Alg_S_O hydrogels, even after one week. In comparison, no gas bubbles were identified for the Alg and Alg_S(15_15) hydrogels.

**Figure 16:**
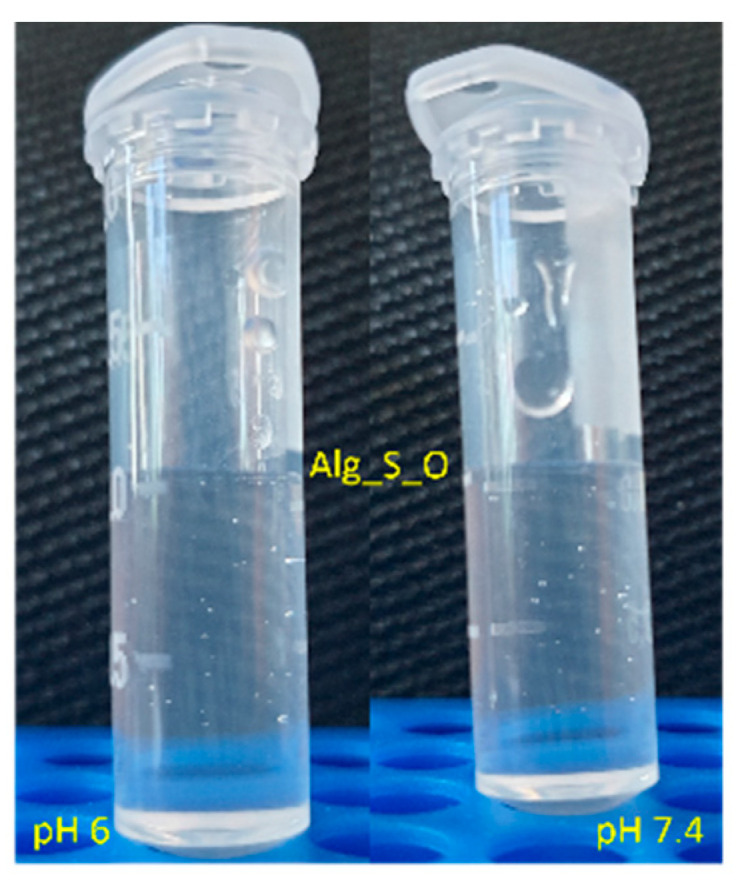
Oxygen bubble visualization for Alg_S_O hydrogel after 10 min in PBS solution at pH 6 (**left**) and pH 7.4 (**right**), revealing their oxygen-releasing ability.

To determine the oxygenation ability of the Alg_S_O hydrogel, its activity under acidic and neutral conditions was evaluated after immersion in PBS solutions with different pH values (6 and 7.4). The apparent pH of healthy skin varies between 4 and 6, depending on the body location, patient’s age, and used cosmetic and cleaning products. The pH of acute skin wounds is neutral, but in the case of bacterial infection, the pH is significantly lower [57,58]. Figure 16 allows for the visualization of oxygen bubbles produced under acidic and alkaline conditions. It was thus evidenced that the Alg_S_O hydrogel had an effective oxygenation activity, being suitable for use in the management of chronic wounds, which undergo hypoxia that ultimately hinders the wound healing process.

**Figure 17:**
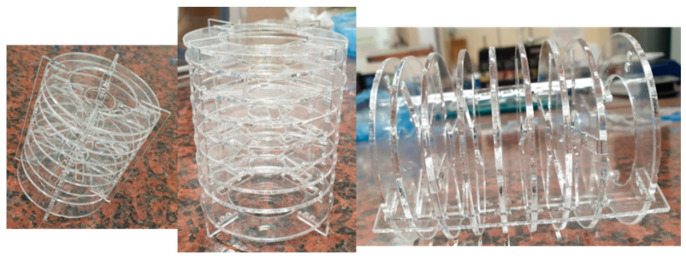
Top-view, front-view, and size-view photos revealing the innovative PMMA discs support configuration for evaluating the real-time H_2_O_2_ release from Alg_S_O hydrogel.

For the qualitative and quantitative analysis of H_2_O_2_, a modified protocol involving the generally known Fenton method, in which a color reaction happens using iron (Fe) as a catalyst and salicylic acid as an organic acidifying compound, was performed. The reaction is based on the formation of complexes between the salicylic acid and FeIII ions, following the H_2_O_2_-mediated oxidation of FeII ions [59,60]. In order to quantify the oxygen-releasing ability of the Alg_S_O hydrogel, the first step was to perform an eight-point calibration curve for H_2_O_2_ (concentration between 0.43 and 17.21 mg/L) after the corresponding spectral measurements in the 350–800 nm wavelength range. A linear result was obtained by plotting the recorded absorbance values versus concentration at the maximal absorption wavelength of 528 nm, with a correlation coefficient (R2) of 0.998. Further, the Alg_S_O sample was tested using a kinetic method, which included the use of innovative support based on nine PMMA discs of 5.8 cm diameter arranged in a shelf-type support (see Figure 17). The PMMA testing architecture, for which the design was conceived with the RDWorksV8 8.01.54 software (Informer Technologies, Inc., Los Angeles, CA, USA), was obtained with a 1610 Pro laser cutting machine (RUBIQ CNC, Bacău, Romania). The PMMA discs were double-sided coated with a thin film of Alg_S_O gel, then introduced into a 300 mL solution of salicylic acid (HOC_6_H_4_COOH) and ferrous sulfate (FeSO_4_ × 7H_2_O) with the addition of acetic acid until it reached an acidic pH of 6. The spectrophotometric measurements were recorded each minute for a total testing time of 500 min.

**Figure 18:**
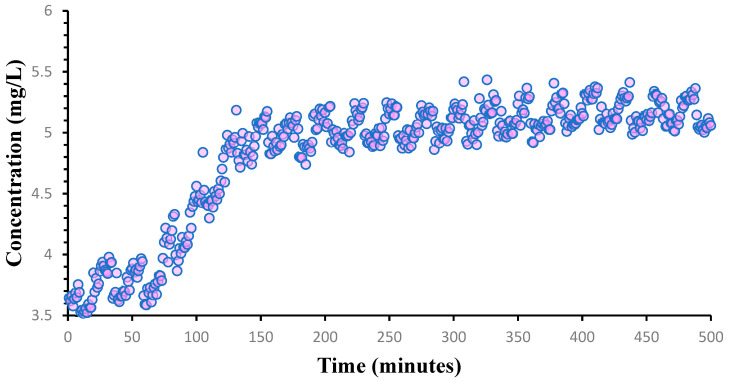
The graphical representation of the UV–Vis results for the Alg_S_O sample, revealing the oxygen concentration expressed in ppm (parts per million) as a function of time (500 min, with every minute recording values).

The equation revealing the absorbance–concentration relation, determined by the standard calibration curve of H_2_O_2_, was used to estimate the concentration (expressed in mg/L) of resulted in H_2_O_2_, which was directly correlated with the time-dependent release of oxygen within the composite hydrogel. The results of the UV–Vis analysis for the Alg_S_O sample are graphically represented in Figure 18.

The UV–Vis results indicated a starting concentration of ~3.5 mg/L of H_2_O_2_ (recorded in the first minute of testing) and an additional increase up to ~4 mg/L in the first hour of analysis. After one hour, a boost in the released concentration of hydrogen peroxide was observed, reaching a maximal value of ~5 mg/L until 2 h of testing. After this time point, a gradual and constant increase in the concentration of released H_2_O_2_ was noticed until the final testing time (~8.5 h), with maximal values that varied between 5–5.5 mg/L.

### 2.4. Cellular Evaluation of Optimized Alginate-Based Hydrogels for Wound Healing Applications

**Figure 19:**
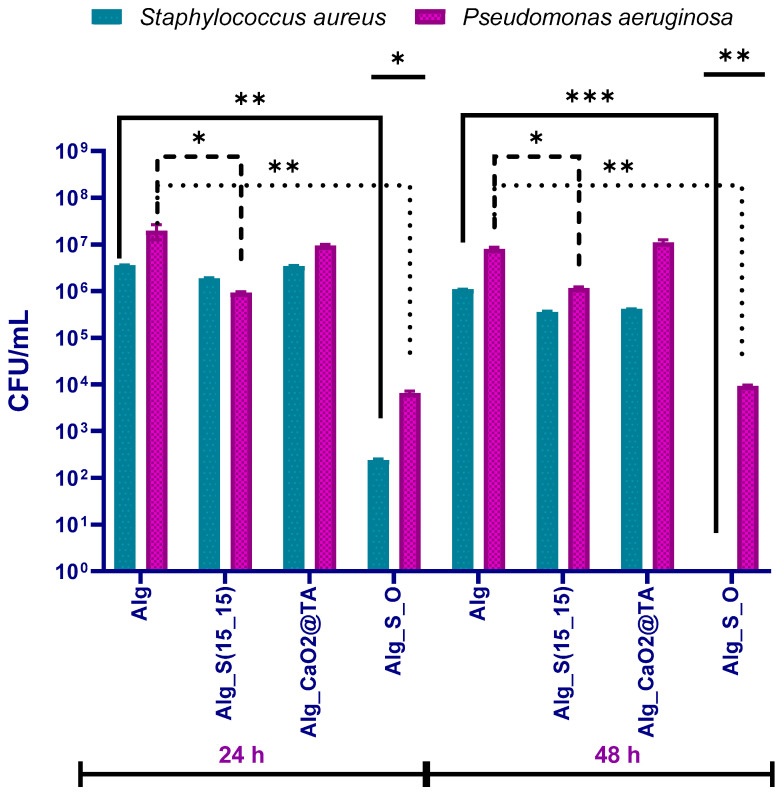
Graphical representation of the biofilm modulation, expressed as log10 CFU/mL, of Gram-positive and Gram-negative bacteria after 24 and 48 h of incubation in contact with the obtained hydrogels. All results represent the mean values of three independent experiments ± S.D. (one-way ANOVA, when comparing samples vs. Alg control * *p* < 0.05; ** *p* < 0.001; *** *p* < 0.0001).

Considering the management of chronic skin wounds by limiting or combating opportunistic infections as an application for the obtained hydrogels, their ability to intercept bacterial contamination and colonization was further assessed. In this regard, bacterial strains that are responsible for the occurrence of wound infections were considered. To evaluate the impact of proposed materials against the development of monospecific bacterial biofilms, the effects of hydrogels were investigated after 24 and 48 h of contact with *S. aureus* and *Ps. aeruginosa*. The data are graphically represented in Figure 19 as log10 CFU/mL values obtained for each sample at the considered testing time points. The antimicrobial effect of nanostructured hydrogel formulations was evaluated by comparison with the Alg sample, which, according to specialized data, lacks an intrinsic anti-pathogenic efficiency.

As a general remark, Alg_S(15_15), Alg_CaO_2_@TA, and Alg_S_O samples had inhibitory effects on both bacterial biofilms after 24 and even after 48 h. In the case of Alg_S(15_15) and Alg_CaO_2_@TA hydrogels, a moderate diminish in the antibiofilm efficiency was observed for both *S. aureus* and *Ps. aeruginosa* strains, when compared to the Alg_S_O sample, which exhibited the most prominent inhibitory action against both bacterial strains at both intervals of evaluation. Moreover, the biofilm inhibition especially occurred for the Gram-positive bacterium, for all samples and particularly after 48 h of incubation in the presence of Alg_S_O hydrogel. Thus, the time required for the complete inhibition of staphylococcal biofilm was 48 h for the hydrogel sample containing both silver nanoparticles and tannylated calcium peroxide nanoparticles. Even if the individual presence of nanoparticles within the alginate matrix determined moderate anti-biofilm effects, synergistic activity was evidenced in the case of Alg_S_O hydrogel, as revealed by the important and preserved ability for reducing or even eradicating the development of bacterial biofilms. These results validated the development of a promising nanostructured hydrogel formulation for wounds prone to infections.

Biocompatibility is unquestionably regarded as a pivotal factor when it comes to developing materials for biomedical applications. In this context, the biocompatibility of the novel materials was investigated using the human HaCaT keratinocytes cell line.

**Figure 20:**
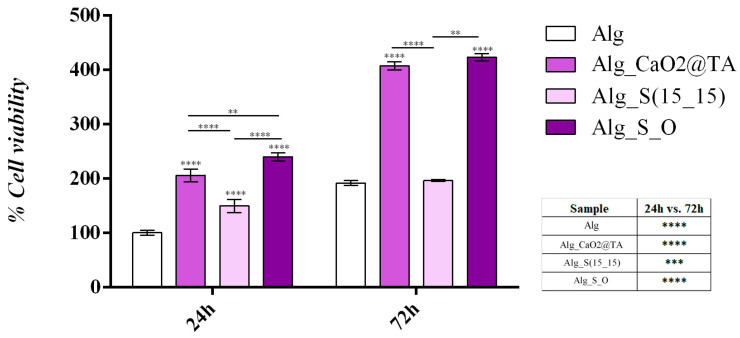
Graphical representation of human HaCaT keratinocytes viability after 24 h and 72 h of contact with the tested samples. The represented data are the mean values of three independent experiments ± S.D. Statistical significance: ** *p* < 0.01; *** *p* < 0.001; **** *p* < 0.0001.

To evaluate the cell viability and proliferation potential of HaCaT keratinocytes following 24 h and 72 h of contact with the Alg-based hydrogel dressings, the MTT assay was performed (Figure 20). The obtained results revealed a statistically significant enhancement in keratinocytes’ viability after 24 h of incubation with all tested samples compared with the Alg control sample, showing that the novel materials better sustained the HaCaT cellular metabolic activity. Similarly, after 72 h of culture, Alg_CaO_2_@TA and Alg_S_O materials induced a statistically significant increase in cell viability, showing consistency in promoting cellular viability over time. However, no noticeable differences in cellular metabolic health were observed for Alg_S(15_15) compared to the Alg control sample after 72 h. Regarding the impact of the Alg-based hydrogel formulations on HaCaT cell proliferation potential, our results indicate that all the tested samples sustained and promoted cellular proliferation, as evidenced by a significant increase in cell viability between the two time-points. Notably, while the Alg and Alg_S(15_15) samples showed a moderate proliferative capacity of HaCaT cells, Alg_CaO_2_@TA and Alg_S_O materials facilitated an increased yield of keratinocyte proliferation.

**Figure 21:**
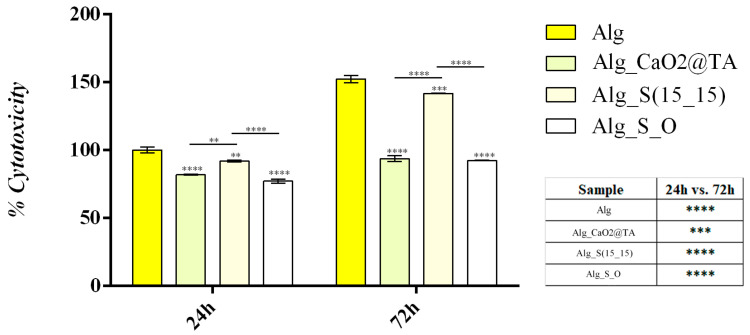
Graphical representation of the LDH leakage in the keratinocyte culture medium after 24 h and 72 h of interaction with the tested samples as a measure of material cytotoxicity. The represented data are the mean values of three independent experiments ± S.D. Statistical significance: ** *p* < 0.01; *** *p* < 0.001; **** *p* < 0.0001.

LDH is an intracellular enzyme released from cells in response to cytotoxic materials that disrupt cellular membrane integrity. Therefore, LDH leakage into the culture medium was measured as an indicator of the cytotoxic potential of the Alg-based formulations (Figure 21). The obtained results were in full accordance with the MTT assay, indicating that the novel Alg-based formulations better sustained the cellular integrity than the simple Alg sample after 24 h, as revealed by the statistically significant decrease in LDH leakage observed in the cell culture medium for all the tested samples compared to the Alg control sample. After 72 h, a similar pattern of LDH leakage was observed in cell culture medium samples collected for the Alg-based formulations compared to the Alg control sample. However, although LDH levels detected in the Alg_S(15_15) sample were lower than those detected in the Alg control, the decrease was moderate compared to the other two tested formulations. For Alg_CaO_2_@TA and Alg_S_O materials, LDH levels were halved compared to the control and Alg_S(15_15) samples, highlighting the cytocompatibility of these samples.

**Figure 22:**
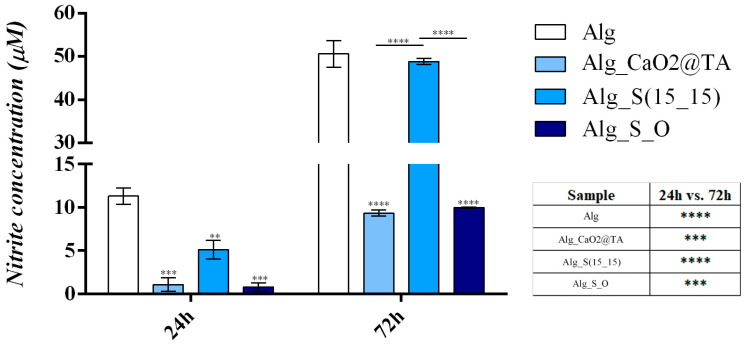
Graphical representation of the nitrite concentration identified in the cell culture medium samples after 24 h and 72 h of HaCaT cell interaction with the tested samples. The represented data are the mean values of three independent experiments ± S.D. Statistical significance: ** *p* < 0.01; *** *p* < 0.001; **** *p* < 0.0001.

Moreover, to assess the potential of the novel Alg-based samples to induce cellular stress and mediate oxidative damage in keratinocyte cultures, the levels of NO_2_^−^ were measured as an indicator of NO production (Figure 22). After 24 h, low levels of NO_2_^−^ were identified in the cell culture medium collected from all Alg-based formulations, the concentration of NO_2_^−^ being statistically significantly lower than the Alg control sample for all tested formulations. After 72 h, the NO_2_ concentration in the cell culture medium collected from Alg_S(15_15) was similar to the concentration of NO_2_^−^ identified in the Alg control sample. In contrast, the levels of NO_2_^−^ in the cell culture medium samples collected from Alg_CaO_2_@TA and Alg_S_O formulations were statistically significantly lower than the levels identified in the Alg control samples. Since low levels of NO production indicate the lack of cytotoxicity of tested materials, the results demonstrate that Alg_CaO2@TA and Alg_S_O formulations do not induce cellular stress in human keratinocyte cell cultures.

**Figure 23:**
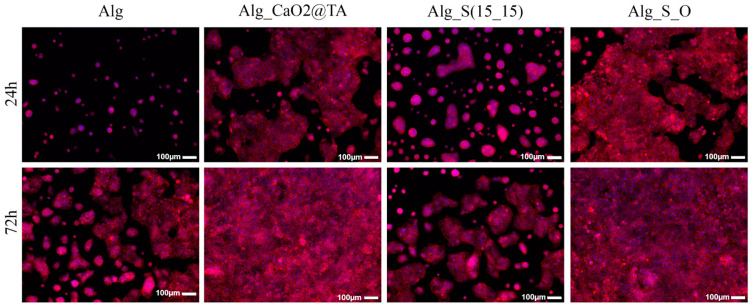
Fluorescence micrographs revealed the human keratinocytes morphology after 24 h and 72 h of culture in contact with the tested samples. F-actin filaments are stained with AlexaFluor 594-phalloidin (red), and cell nuclei with DAPI (blue). (10× magnification).

To reveal the potential of Alg-based materials to trigger morphological changes in HaCaT cells, the expression of F-actin filaments was investigated through fluorescence microscopy (Figure 23). After 24 h of culture, the human keratinocytes cultured in contact with the Alg and Alg_S(15_15) samples exhibited a rounded morphology with a poorly developed cytoskeleton, forming small cell clusters. In contrast, cells cultured on Alg_CaO_2_@TA and Alg_S_O samples adapted their typical polygonal shape, accompanied by a well-developed cytoskeleton and a dynamic network of F-actin filaments distributed throughout the cytoplasm. Increasing the culture time to 72 h sustained the HaCaT cells to adopt their typical morphology on the Alg (control) and Alg_S(15_15) samples. However, when in contact with the Alg_CaO_2_@TA and Alg_S_O formulations, the HaCaT cells developed significantly more complex 3D cellular networks that fully covered the sample surfaces, with keratinocytes presenting a cytoskeleton rich in F-actin filaments.

## 3. Discussions

The evolution of silver nanoparticle synthesis receives the attention of the scientific community due to the peculiar properties exhibited by the nano-silver-based materials. Considering the size-related unique characteristics of silver nanoparticles, together with their extensive anti-pathogenic efficiency, various ready-to-use products are available or under commercial implementation for contemporary applications, including pharmaceutical and cosmetic products, anti-infective coatings for medical devices, wound dressings and antimicrobial textiles, food packaging, and platforms for environmental remediation [61,62]. With respect to their biomedical applications, the focus is on developing new facile, fast, and efficient methods to synthesize these nanoparticles, with particular attention towards the high-yield and reproducible fabrication of particles with tuned outcomes. In this sense, the microfluidic approach provides a fine line for obtaining nanoparticles with controlled physicochemical properties through the involvement of some inexpensive equipment and a lab-on-chip platform that provides optimal conditions for the safe and self-sustained production of nanoparticles [63,64].

In this study, silver samples have been synthesized under the control of small volumes of AgNO_3_–PVP and ascorbic acid solutions through an optimized cross-shape chip by varying the input parameters (admission speed expressed in RPM) of reactants. Ascorbic acid is a potent reducing agent of silver ions, enabling the formation of nano-silver with different morphologies [65,66], while poly(vinyl pyrrolidone) (PVP) acts as a strong stabilizing and dispersant agent for silver nanostructures [67,68]. Herein, XRD results indicated the formation of highly crystalline silver samples, consisting of nanosized crystallites (~21 nm) and a sole crystalline phase, whose identification through Miller indices corresponded to cubic silver (Figure 1, Table 2). Following a comparative study between different synthesis methods of nano-silver in the presence of ascorbic acid and PVP, Chen et al. [69] reported an increased crystallinity of the flower-like silver obtained using an S-shaped microfluidic device compared to the sphere-like particles synthesized through a conventional reduction method. The morphology of synthesized silver samples was evaluated through SEM and TEM analysis (Figure 2 and Figure 3), which revealed the sphere-like arrangement of constituent silver nanoparticles. Similarly, the formation of sub-micron silver nanostructures with sphere or star morphology has been reported by using higher or lower concentrations of PVP, respectively, while maintaining a constant AgNO_3_/ascorbic acid ratio [70]. The spherical shape of silver samples is due to the selection of both reducing (ascorbic acid) and stabilizing (PVP) agents. In our previous study, quasi-spherical silver nanoparticles with a high agglomeration tendency were obtained by using the same chip design, the same admission speed of reactants (RPM), and the same concentration of AgNO_3_, but employing D-glucose as a reducing agent [71]. Moreover, the concentration of metallic precursor (AgNO_3_) and reducing/capping agents (ascorbic acid and PVP) is critical in creating the desired morphology. For instance, the formation of spherical silver nanostructures was correlated with the use of higher concentrations of low-molecular PVP or lower concentrations of high-molecular PVP [68,72]. Still, distinctive morphologies were also reported by varying other reaction parameters (stirring time, millisecond-tuned vortexing, temperature, irradiation) [65,73,74,75]. The role of PVP is significant because it stabilizes the silver nanoparticles through intramolecular bonds with the metallic atoms and the formation of an outer layer over the particles, thus generating an electrostatic shield that decreases the probability of agglomeration. The formation of silver microspheres is very likely to have occurred due to the concurrent use of PVP and ascorbic acid, as the polymer may dictate the direction and speed of nanoparticles’ growth, while the antioxidant concentration influences the sphere-like morphology of particles. A study by Qin et al. [74] revealed that the utilization of ascorbic acid for reducing silver preferentially directed towards forming single particles with quasi-spherical shapes. Chen and co-workers [69] also tested the influence of ascorbic acid and PVP concentration in obtaining silver nanoparticles through a microfluidic device. By using 0.02 M AgNO_3_, 0.2% PVP, and 0.01 M ascorbic acid, they reported the formation of flower-like silver nanoparticles, while particles with a sphere-like morphology resulted by using the same AgNO_3_ concentration with 1% PVP and 0.1 M ascorbic acid. Higher PVP concentrations induced a bridging process between nanoparticles, promoting their sphere-like agglomeration. Our results line up with other findings on the microfluidic synthesis of silver nanostructures, pointing out a complicated kinetic pathway in which early crystallization that occurs during the formation of inorganic nanoparticles in the presence of a stabilizing agent allows for a better control over their size and shape [38,75,76,77]. Also, this could indicate that the formation of silver nanostructures was principally mediated by the aggregation of small agglomerations of nanoparticles, and not by the possibility of coalescence which is characteristic for larger particles [78,79,80,81]. DLS analysis revealed important aspects about the silver samples, evidencing distinctive behaviors for all four silver nanostructures when in contact with aqueous media (Figure 4). S(15_15) silver spheres were identified as the most stable sample according to the hydrodynamic diameter and zeta potential measurements, with corresponding values of 370 nm and −7.62 mV, respectively. The explanation behind the DLS results was related to the influence of PVP. The PVP outer layer determined a particular assembly of the silver nanoparticles in sub-micron-sized spheres which led to increased average hydrodynamic sizes in comparison with physical dimensions of silver samples. The larger the hydrodynamic diameter average, the more difficult it will be to maintain stability. However, the same PVP layer had an effect on the surface charge of silver samples by decreasing the zeta potential. The influence of PVP on increasing the stability of silver nanostructures by forming an outer electrostatic shield able to prevent or limit inter-particle interactions was observed in various studies, and we therefore concluded that the S(15_15) silver is relatively stable and presents suitable properties for our intended application [82,83,84,85,86].

Calcium peroxide nanoparticles represent a simple and effective candidate for overcoming low oxygen levels and opportunistic infections found at the chronic wound site, which hinder the healing process and may result in moderate-to-severe complications. In this study, we synthesized CaO_2_ nanoparticles by precipitation, which provides a low-cost, simple, and tunable process for obtaining particles with controlled outcomes. Tannic acid (TA) was used as a stabilizing agent, but it also enabled the obtaining of CaO_2_ nanoparticles in non-special temperature conditions while possessing an active role in the treatment of wounds [47,87]. The XRD data confirmed the presence of CaO_2_ as a single phase (Figure 5), with a mean crystallite size of 47.34 nm. Sapana and colleagues [88,89] synthesized CaO_2_ nanoparticles using a precipitation method starting from CaCl_2_ and using polyethylene glycol (PEG) as a stabilizing agent. They reported average particle sizes of 16.8 nm and 16 nm and reduced samples’ crystallinity due to the presence of polymer. Shen et al. [90] developed a self-activating hydrogel using CaCl_2_ and PEG, and the collected diffraction pattern showed that CaO_2_ was the only crystalline phase of generated nanoparticles. Considering the FTIR results in our study (Figure 5), the successful synthesis of tannylated calcium peroxide particles was proven. Specific vibrations of the O–Ca–O functional group were identified only in the CaO_2_@TA sample (~517 cm^−1^), together with the stretching modes of peroxide (~780 and ~863 cm^−1^) [91,92]. B.S. De et al. [93], Li F. et al. [94], and Sajedeh K. et al. [95] synthesized calcium peroxide nanoparticles in their studies and demonstrated the presence of O–Ca–O stretching in FTIR spectra in the 508–590 cm^−1^ wavenumber interval. The presence of tannic acid in the CaO_2_@TA sample was given by the conjugated carbonyl moieties, identified at ~1700 and 1595 cm^−1^ wavenumbers [55,56]. Also, the ester C–O group was observed at ~1321 and ~1075 cm^−1^, which corresponded with the findings reported by Hayat et al. [96] and Wei et al. [97]. The morphological characteristic of calcium peroxide nanoparticles is their spherical shape. We developed CaO_2_@TA nanoparticles that fulfilled this morphological particularity, as evidenced by the electron microscopy results (Figure 6 and Figure 7). Well-defined individual particles with round shapes, mean particle size below 150 nm, and a relative agglomeration tendency have been obtained in our study. The polycrystalline nature is another particular feature of calcium peroxide nanoparticles, which also applies for the synthesized CaO_2_@TA sample. Our results on the tannylated nanoparticles are compliant with those of other studies. For instance, Guowen and co-workers [98] obtained polycrystalline calcium peroxide particles (2.5 μm) using a hydrolyzation–precipitation protocol, then used them as nucleation platforms to develop pH-responsive constructs that were finally loaded within polyester scaffolds for the local management of bone tumors. Zhiyong Z. et al. [99] synthesized CaO_2_ nanoparticles (sizes between 80 and 150 nm) to be used as nano-fillers in nanofiber films for wound healing, while Yanyan Y. et al. [100] utilized nano-CaO_2_ (100–150 nm particle size) as an ion interference strategy for calcium overload. Hydrodynamic diameter (nm) and zeta potential (mV) characteristics were also evaluated for the CaO_2_@TA sample (Figure 8). The mean value of the hydrodynamic diameter (393.2 nm) was almost double the physical size measured from the micrographs, according to the theory regarding the size of the particles in liquid. The increased stability of the sample was correlated with the measured values of zeta potential (−16.48 mV) along with the negative surface charge of CaO_2_@TA nanoparticles. Our findings are in agreement with the results of Yanyan et al. [100] and Shuangrong et al. [101], where zeta potential values of −15.8 mV and −10.8 mV, respectively, were reported. Compared to their results, the highly abundant hydroxyls and carboxyl ions within the TA led to a more negative surface charge, finally resulting in a better stability of CaO_2_@TA nanoparticles.

This study aimed to obtain biocompatible dressing formulations that could solve two critical problems at the level of chronic wounds, namely infection and hypoxia, both being strongly damaging factors for the wound healing process. Silver nanoparticles, which are recognized for their strong and extended anti-pathogenic efficiency [100,101,102,103,104,105,106], were obtained through a microfluidic method to combat the infection. Following their characterization, a single synthesized silver sample (S_(15_15)) was selected to be used in the development of dressings. Given their impressive and favorable role in the release of oxygen at the wound level [44,106,107,108], tannylated calcium peroxide nanoparticles (CaO_2_@TA) were synthesized and investigated in the prospect of enhanced wound healing. When CaO_2_ nanoparticles are exposed to a moist environment, the oxygen generation process occurs following a decomposition reaction [109,110]. One important fact is the capacity to control the slow release of small oxygen concentrations, considering that severe oxidation stress may appear [7]. We visually examined the ability of tannylated nanoparticles to generate oxygen bubbles (Figure 9), and the collected images revealed a time-dependent and pH-mediated process, which resulted in accelerated oxygen bubble formation under acidic conditions. To obtain the dressing formulations, sodium alginate was used as a polymer matrix in the structure in which the nanoparticles were embedded. Four hydrogel formulations were considered: alginate (Alg), silver-modified alginate (Alg_S(15_15)), calcium peroxide-modified alginate (Alg_CaO_2_@TA), and alginate modified with S(15_15) and CaO_2_@TA nanoparticles (Alg_S_O). The comparative FTIR analysis mostly revealed alginate-originating moieties, as the polysaccharide represented the major component of all hydrogel formulations (Figure 10). Three significant differences were observed between all four FTIR spectra, starting with a decrease in the absorbance of hydroxyls (3050–3500 cm^−1^) after embedding the CaO_2_@TA nanoparticles (Alg_CaO_2_@TA and Alg_S_O), due to alginate–Ca^2+^ interactions. The second one was that Alg_S(15_15) and Alg_CaO_2_@TA samples exhibited the highest and lowest absorbance bands, respectively. This outcome might have congruently resulted from the presence of PVP-stabilized silver (leading to additive hydroxyls and conjugated carbonyls, and superposition of amide vibrations over C–O bands) and Ca^2+^-enriched nanoparticles (leading to physically cross-linked alginate networks) [111,112]. The last observation was the distinctive presence of the O–Ca–O functional group in the spectral results of the Alg_CaO_2_@TA and Alg_S_O samples. The selected polymeric matrix fulfills the biocompatibility and biodegradability requirements of an ideal dressing, along with a porous morphology. SEM micrographs showed the obtained materials’ organized and interconnected porous structure (Figure 11), with similar pore shape and size for the CaO_2_@TA-free hydrogels. By contrast, a close-packed porosity, with much smaller pores, was observed for the CaO_2_@TA-embedded samples, with average pore dimensions of 25.97 ± 0.83 µm (Alg_CaO_2_@TA) and 31.50 ± 0.81 µm (Alg_S_O). This outcome was consistent with the FTIR results, being particularly related to the Ca^2+^-mediated reticulation of the alginate matrix. The SEM data and EDS results (Figure 12 and Figure 13) revealed the uniform distribution of S(15_15) spheres and CaO_2_@TA aggregates within the polysaccharide matrix.

The results obtained by evaluating the swelling behavior of hydrogel dressings over a period of 96 h indicated their increased swelling efficiency, with rates over 100% being determined for all samples (Figure 14). Using sodium alginate co-doped with calcium peroxide and oxidized mesoporous carbon nanoparticles along with L-arginine, Zhang et al. [90] developed a self-activating NO-releasing hydrogel with porous morphology and a swelling rate of 61.5% after 24 h. The Alg_S(15_15) hydrogel exhibited the lowest swelling rate, most probably due to the presence of S(15_15) nanoparticles, which either prevented the liquid passage by obstructing the alginate pores or limited the hydration of the alginate matrix due to increased interactions between nanoparticles and polymer chains. Similar outcomes, in terms of increased porosity and decreased swelling rate, have been previously reported for alginate-based hydrogels loaded with silver nanoparticles [113,114]. The Alg_CaO_2_@TA hydrogel displayed the highest swelling rate (159%) and lowest degradation rate (15%), as evidenced after 4 and 14 days of testing, respectively. The latter outcome was associated with the reticulation effect of calcium ions on the polysaccharide matrix and consequent denser porosity.

Regarding the swelling behavior of this sample, the release of TA molecules might have resulted in a local acidic environment, which determined strong electrostatic repulsions between constituent carboxyl ions and further facilitated the diffusion of water molecules into the polysaccharide network [115,116]. Similar swelling rates were evidenced for Alg_S_O (129%) and Alg (132%) hydrogels, the results achieved for the three-component sample being compliant with previous observations on the individual effects of silver and tannylated calcium peroxide nanoparticles on the swelling behavior of alginate hydrogels. The Alg_S_O sample exhibited the highest degradation rate (30%), which was correlated with the simultaneous presence of silver nanoparticles (that could have mediated the degradation process either by migrating from the polymeric matrix to the SBF medium in time or by the PVP hydrolysis) and CaO_2_@TA nanoparticles (that could have influenced the degradation process by the local release of TA).

When tested under bio-simulated neutral conditions for one week, CaO_2_@TA-loaded hydrogels (Alg_CaO_2_@TA and Alg_S_O) displayed the ability to generate oxygen bubbles (Figure 15), which was consistent with previous observations regarding a delayed gas bubble formation that has been evidenced for tannylated nanoparticles after 5 days (Figure 9). Further evaluation of Alg_S_O hydrogels under acidic and neutral conditions revealed their effective oxygenation activity (Figure 16), while spectrophotometric analysis was performed to quantitatively evaluate the corresponding oxygen release, by measuring the levels of hydrogen peroxide up to 8 h (Figure 18). The presence of H_2_O_2_ was proven through a colorimetric assay using salicylic acid and iron (II) sulfate heptahydrate, where purple solutions resulted from increasing H_2_O_2_ levels. Compliant with the increased ability of tannylated nanoparticles to induce oxygen bubble formation under acidic environments (Figure 9), UV–Vis results indicated a stabilization stage in the first hour, followed by a burst release of H_2_O_2_ in the next hour of evaluation (Figure 18). Further, a slow and gradual increase in the concentration of released H_2_O_2_ was noticed until the final testing time (~8.5 h), with a maximal released value of ~5.5 mg/L that was equivalent to 10.8 µM, which indicated a controlled release with a safe effect regarding the oxygenation. Pan et al. [117] reported the beneficial role of low H_2_O_2_ concentrations on epithelial cells. At 24 h post-exposure, they showed enhanced migration and stimulated proliferation in cells treated with 10 and 20 µM H_2_O_2_, while exposure to 60 µM H_2_O_2_ reduced the cell viability. Lee et al. [118] reported the synthesis of hydrogels with different feed amounts of H_2_O_2_ (1, 2.5, 5, and 10 mM) for the treatment of drug-resistant bacterial infections. The kinetic release study of H_2_O_2_, performed up to 20 h using a spectrophotometry method, revealed that 2 µM and 509 µM were the smallest and highest concentrations of released H_2_O_2_ (obtained for hydrogels obtained with 1 mM and 10 mM amounts of H_2_O_2_, respectively). Moreover, they demonstrated that the hydrogel formulation synthesized with a 1 mM H_2_O_2_ amount (corresponding to 2 µM released hydrogen peroxide) slightly increased the viability of human keratinocytes, whereas the hydrogel with a 10 mM H_2_O_2_ amount decreased the viability in both keratinocyte and fibroblast cell cultures. Our results evidenced an effective maximal release of 10.8 µM H_2_O_2_ after 8 h, which was in agreement with other studies reporting that low concentrations of H_2_O_2_ stimulate normal processes in healthy cells, ultimately contributing to an enhanced wound healing process. 

To evaluate the antibiofilm effects of the hydrogels, two representative bacterial strains (*S. aureus* and *Ps. aeruginosa*) were used at two different times of incubation (24 and 48 h). The data were represented as log10 CFU/mL (corresponding to the amount of biofilm-forming viable cells) for each sample (Figure 19). All hydrogel formulations displayed inhibitory effects against both bacterial biofilms after both testing intervals, but the Alg_S_O sample exhibited the most prominent inhibitory action. The most evident result was the significant antibiofilm activity of tested samples against the Gram-positive strain, especially in the case of the Alg_S_O sample, which determined a total inhibition of the staphylococcal biofilm after 48 h. A valid explanation for this outcome is the synergistic action of silver and calcium peroxide nanoparticles against the Gram-positive bacteria, which implies different antimicrobial mechanisms that depend on the bacterial cell membrane. The peptidoglycan-based cell wall of the Gram-positive bacteria exhibits a multilayered structure and an increased thickness in comparison with the Gram-negative cells, which possess a thinner peptidoglycan layer and an outer lipopolysaccharide membrane. Even if the expectations were directed towards a better effect against the Gram-negative strain, the more compact ultrastructure of the peptidoglycan layer and the lower permeability of the outer membrane provided an active barrier against the nanoparticle-medicated destabilization and piercing of bacterial cells. Moreover, the stronger antibiofilm efficiency against the Gram-positive bacteria was supported by the characteristic increased porosity and negatively charged surface, which contributed to the inveiglement of the positively charged silver and calcium ions [119,120,121]. Compared to the unmodified Alg material, the CaO_2_@TA-loaded hydrogel determined only a slight decrease in *Ps. aeruginosa* populations after 24 h but more prominent inhibitory action against the staphylococcal biofilm at both time points (being more effective after 48 h). This effect might have resulted from the concurrent action of calcium peroxide nanoparticles and tannic acid, whose main antibacterial mechanisms include oxidative stress and disruption of the bacterial metabolism, respectively [47,122]. Our results are in accordance with the study made by Hong Yu et al. [123], who proposed the fabrication of sprayed polyacrylic acid, calcium peroxide nanoparticles for the treatment of wound infection, as their microbiological results revealed a higher efficiency against the Gram-positive *S. aureus* compared to the Gram-negative *E. coli*. A moderate antibiofilm efficiency was evidenced for the Alg_S(15_15) hydrogel on both pathogens at both experimental times but with more prominent inhibitory effects against the Gram-negative strain. This outcome was in good accordance with other studies that reported the anti-pseudomonal efficiency of nano-silver-embedded alginate composites for wound healing applications [124,125]. The less negatively charged S(15_15) silver (compared to tannylated nanoparticles) and much thinner peptidoglycan layer of *Ps. aeruginosa* could have collectively determined an accelerated cellular uptake of silver nanostructures. After internalization of nano-silver, Ag^+^-mediated oxidative damage, together with Ag^+^-altered cellular metabolism and survival, generally occurs in pathogenic cells [126,127]. Our results showed that the best biofilm inhibition occurred for the Alg_S_O hydrogel, regardless of the bacterial strain or testing time (Figure 19). When compared to bare Alg samples, an important and sustained inhibition of the pseudomonal biofilm, with a preserved efficiency of three logs, was evidenced after 24 and 48 h. In the case of *S. aureus*, Alg_S_O hydrogels determined a prominent reduction in bacterial populations with more than four orders of magnitude (four logs) after one day while causing total biofilm eradication after 48 h. We related this outcome to the synergistic antibacterial effects of silver and tannylated nanoparticles, particularly emphasizing the bactericidal role of the reactive oxygen species (ROS). Though both selected nanoparticles exhibit ROS-mediated antimicrobial effects, silver nanoparticles’ efficiency depends on the presence of oxygen. Since Alg_S(15_15) hydrogels exhibited a moderate antibiofilm efficiency, validating previous data on the intrinsic anti-pathogenic activity of nano-silver, this theory was herein supported by the oxygenation ability of CaO_2_@TA nanoparticles and CaO_2_@TA-loaded hydrogels. This concept was tested by Xu et al. [128], who reported a significantly higher antimicrobial activity of silver nanoparticles against *E. coli* under aerobic conditions compared to anaerobic ones, thus revealing that ROS production had an important role in the efficacy of silver nanoparticles against microorganisms. Moreover, the results of Kora and co-workers [129] supported the ROS-mediated damage and cell surface injury of bacterial strains under the activity of silver nanoparticles. Using H_2_O_2_ as a positive control, they reported higher levels of intracellular ROS after exposure to nano-silver, with moderate oxidative stress against Gram-negative strains. Other observations of this study were the tolerance of *Ps. aeruginosa* towards the activity of H_2_O_2_, attributed to the shielding role of the basic expressed catalase enzyme, and the highest intracellular ROS generation that occurred in *S. aureus* [130]. Concluding our results, the oxygen-releasing tannylated calcium peroxide nanoparticles had a potentiating effect on the antimicrobial activity of silver nanoparticles, resulting in a significantly improved antibiofilm efficiency of Alg_S_O hydrogels against Gram-positive and Gram-negative bacterial strains, and even the complete eradication of the staphylococcal biofilm after 2 days.

The biological evaluation of the obtained hydrogels was assessed on HaCaT keratinocytes, starting with the MTT assay that was performed after 24 and 72 h of contact in order to analyze the cell viability and proliferation potential of the proposed hydrogels (Figure 20). Alg_S(15_15) hydrogels determined a statistically significant increase in the viability of HaCaT cells only after 24 h compared to Alg, while no noticeable differences in the metabolic activity of keratinocytes were observed for these samples after 3 days of incubation. This result was merely correlated with the released S(15_15) silver, which determined a progressive cell membrane impairment and oxidative damage (Figure 21 and Figure 22, respectively). Similar outcomes have also been reported for silver-containing Alg-based commercial dressings due to the increased sensitivity of keratinocytes towards nano-silver [131]. Compared to Alg hydrogels, both Alg_CaO_2_@TA and Alg_S_O formulations presented an important and prolonged ability to sustain and promote cell viability (Figure 20) and support cellular integrity (Figure 21), while they did not induce oxidative stress in human keratinocytes (Figure 22). These findings were particularly assigned to the presence of tannylated calcium peroxide nanoparticles with oxygen-releasing ability, with a gradually released H_2_O_2_ concentration of 10.8 µM after ~8.5 h. The cellular assays demonstrated the safe oxygenation occurred in HaCaT cells when incubated on CaO_2_@TA-loaded hydrogels, which lined up to previous studies regarding the beneficial role of low H_2_O_2_ concentrations on epithelial cells, fibroblasts, and keratinocytes [117,118]. Also aiming for the management of hypoxic skin wounds, CaO_2_-loaded hydrogels based on different polysaccharides (which possess an intrinsic moisture retention ability and exert beneficial bioactivity with respect to tissue repair and regeneration, such as alginate, chitosan, hyaluronic acid) have been validated as safe dressing candidates for the uncomplicated and accelerated wound healing [106,132,133]. Using different amounts of CaO_2_ nanoparticles, M.Y. Mollajavadi et al. [134] developed an alginate-based scaffold with a sustained oxygen-releasing ability (>5 mg/L for 1 week). The cell viability results, evaluated after 1, 3, and 7 days of contact with fibroblast cells, revealed the best outcome for the Alg sample with 1% (*w*/*w*) calcium peroxide nanoparticles after every time of evaluation, while a decrease in the cellular viability was observed for hydrogels with higher amounts of calcium peroxide (5%). Adding CaO_2_ at 8–12 mg/mL concentration within gelatin/hyaluronan matrix enabled the fabrication of sprayable hydrogel dressings with prolonged oxygen-releasing capacity (2 weeks), antibacterial properties, and an important ability to diminish the metabolic stress and reduce cell death in hypoxic fibroblasts [135]. Given their extended-release oxygen profile, CaO_2_-loaded polyester microspheres (either solitary or encapsulated within a self-polymerized peptide hydrogel) exhibited long-term beneficial effects on normal cells [136,137]. An oxygen-generating implantable material consisting of alginate beads encapsulating CaO_2_ and catalase has been developed by Huang and colleagues [138] as an active carrier for doxorubicin, with the aim to increase the drug sensitivity in cancer cells undergoing hypoxia-induced chemo-resistance. When assessed on human hepatoma cell lines under hypoxic and normoxic conditions, similar viability was reported for Alg-based pellets upon a 20 pellets/mL concentration, compared to control specimens. Still, potentiated ROS-induced cytotoxicity was observed for cancerous cells concomitantly treated with doxorubicin and CaO_2_ nanoparticles. Another study [139] evaluated the viability of cardiomyocytes after 10 days in contact with a polyurethane scaffold embedding calcium peroxide. All materials, with oxygen release activity ranging from ~8 mg/L (1 day) to ~3–4 mg/L (10 days), sustained the percentage of viable cells cultured under hypoxic conditions close to that of the control (polyurethane) samples in normoxic cultures, for up to 10 days. Pedraza et al. [140] synthesized a long-term oxygen-generating biomaterial based on polydimethylsiloxane (PDMS) and calcium peroxide and evaluated its behavior in pancreatic β cell cultures. After overnight incubation in contact with the PDMS_CaO_2_ material, the results on hypoxic cells revealed a benefic influence from the oxygen-generating material with respect to viable cells, with significantly increased MTT levels. Also, a moderate increase in cell viability was observed for the normoxic cells. The LDH assay, performed in the same conditions (hypoxic and normoxic), showed a significant decrease in the LDH levels released by cells in contact with the PDMS_CaO_2_ material under both oxygen conditions. In our study, the collected results revealed the lack of toxicity of nanostructured hydrogel formulations in HaCaT cell cultures, with the lowest levels of released LDH being evidenced for the Alg_S_O sample after 72 h (compared to unmodified Alg hydrogel). These results were in concordance with the MTT assay data, which indicated the superior cell survival and proliferative potential of keratinocytes in contact with the Alg_S_O formulation, especially. Moreover, the Alg_S_O material promoted and supported the normal development of HaCaT cells (Figure 23), allowing for their attachment and well-developed cytoskeleton starting from a short incubation time, and for their uniform spreading and formation of complex cellular networks after 3 days. Collectively, the biological results demonstrated the high biocompatibility of alginate hydrogels embedding silver and tannylated calcium peroxide nanoparticles with respect to human keratinocytes, validating the potential of Alg_S_O formulation for the management of hypoxic wounds.

## 4. Materials and Methods

### 4.1. Materials

Analytical-graded reagents were purchased from Sigma-Aldrich/Merck (Darmstadt, Germany) and used without additional purification to synthesize the materials of interest. The following reagents were considered: silver nitrate (AgNO_3_), ascorbic acid (C_6_H_8_O_6_), poly(vinylpyrrolidone)—PVP (C_6_H_9_NO)_n_, calcium chloride dihydrate (CaCl_2_ × 2H_2_O), tannic acid—TA (C_76_H_52_O_46_), absolute ethanol (C_2_H_6_O), ammonium hydroxide (NH_4_OH), hydrogen peroxide (H_2_O_2_), and sodium alginate. All solutions required for synthesis were prepared using distilled water. The provider of cell culture chemicals and specific kits used during (micro)biological assays were mentioned in the protocols accordingly.

### 4.2. Synthesis Methods

#### 4.2.1. Synthesis of Silver (S) Nanoparticles

A microfluidic method for obtaining silver nanoparticles was approached using a PMMA-based (poly(methyl methacrylate)) platform manufactured with a 1610 Pro laser cutting machine [71,141]. In this sense, a cross-shaped platform with a central inlet for AgNO_3__PVP solution (mixture of 0.03 M silver nitrate and 1% poly(vinylpyrrolidone) and two side inlets for 0.2 M ascorbic acid solution was used. Freshly prepared reactant solutions were introduced in the microfluidic platform using three-stop tubes, then operated and controlled using a peristaltic pump with four channels and twelve rollers. For process optimization, four silver-based batches were obtained by varying the input of AgNO_3__PVP and ascorbic acid solutions. The flow rate (indirectly estimated by the number of rotations per minute—RPM—on the peristaltic pump channels) of metallic and reducing solutions was considered for sample coding, as presented in Table 2.

#### 4.2.2. Synthesis of Tannylated Calcium Peroxide (CaO_2_@TA) Nanoparticles

To obtain calcium peroxide nanoparticles, the precipitation method was used. CaCl_2_ × 2H_2_O and TA (10:1 wt%) were dissolved in ethanol (7.3 mg/mL concentration) using an ultrasound bath, then the solution was stirred at 600 RPM. This time, NH_3_ 1 M was added to the ethanolic solution (0.05:1 *v*/*v*), and the mixture was allowed to react for 2 min. The next step involved the reaction with H_2_O_2_ solution by gradually adding (every 2 min, 16 times) of 100 µL of H_2_O_2_ 35% solution. A final sonication process took place for 10 min, and then the complete reaction occurred overnight at room temperature. The resulting particles were centrifuged for 10 min at 6000 RPM, and three successive centrifugation washing processes with 99% ethanol were carried out, followed by overnight drying at 60 °C.

#### 4.2.3. Synthesis of Alginate-Based Hydrogels Embedding S(15_15) and CaO_2_@TA Nanoparticles

All hydrogel formulations implied the use of sodium alginate solution at a concentration of 5 wt%, as the polymeric matrix. The first synthesized hydrogel consisted of sole alginate, which was considered the control and coded as Alg. The second hydrogel involved the addition of an optimized silver sample, as identified through the physicochemical results. The S(15_15) sample was dispersed in 200 µL of deionized water under sonication and then added to the polymeric matrix. Alg_S(15_15) was the sample code for the silver-decorated alginate hydrogel, in which components were mixed in a 1:1 (*v*:*w*) ratio. In a similar way, the third hydrogel, coded Alg_CaO_2_@TA, consisted of alginate and CaO_2_@TA in a 1:1 (*v*:*w*) ratio. To obtain a wound dressing formulation that has antimicrobial properties and provides oxygen in chronic wounds, the fourth hydrogel—referred to as Alg_S_O—consisted of sodium alginate, silver nanoparticles (S(15_15)), and tannylated calcium peroxide nanoparticles (CaO_2_@TA) in 1:1:1 (*v*:*w*:*w*) ratio. The synthesis of alginate-based hydrogels considered the physical cross-linking method mediated by ionic interactions between the cation (Ca^2+^) and the carboxyl functional groups of the polymer chains. The source of Ca^2+^ was conventional calcium chloride (CaCl_2_) solution (2 wt% concentration) or calcium peroxide (CaO_2_@TA nanoparticles). Regardless of the cross-linking agent (CaCl_2_ for Alg and Alg_S(15_15) samples, and CaO_2_@TA for Alg_CaO_2_@TA and Alg_S_O samples, added in 1:1 (*v*:*v*) and 1:1 (*v*:*w*) ratio, respectively), the final hydrogels resulted after 1 h of continuous stirring at room temperature, with an extra 10 min of stirring for the distribution of S(15_15) silver within the polymer matrix (Alg_S(15_15) and Alg_S_O).

### 4.3. Physicochemical Characterization Techniques

#### 4.3.1. X-ray Diffraction (XRD)

The crystallinity of the powdery samples obtained through microfluidic (silver-based) and precipitation (calcium peroxide-based) synthesis was checked using a PANalytical Empyrean diffractometer (PANalytical, Almelo, The Netherlands) supplied with a (2xGe 220) hybrid monochromator on the incident side and a parallel plate collimator mounted on a PIXcell 3D detector on the diffracted side. The measurements were performed at room temperature by means of Grazing Incidence X-ray Diffraction (GIXRD), with the following parameters: angle of incidence ω = 0.5° for Bragg angles of 2θ between 10° and 80°. The diffractometer used Cu_Kα_ radiation, with λ = 1.5406 Å (40 mA and 45 kV).

#### 4.3.2. Scanning Electron Microscopy (SEM)

The morphological and dimensional characteristics were evaluated using an Inspect F50 scanning electron microscope coupled with an energy-dispersive spectrometer (EDS), purchased from Thermo Fisher Scientific (former FEI, Eindhoven, The Netherlands). Using a carbon-bearing strip, the powdery samples were fixed and inserted into the analysis chamber of the microscope. The same sample preparation process was also used for the hydrogels after they were covered with a thin layer of gold (aimed to reduce electric charges). The micrographs were acquired using 30 keV secondary electron beams.

#### 4.3.3. Transmission Electron Microscopy (TEM)

TEM analysis was performed using high-resolution 80–200 Titan Themis equipment with a selected area electron diffraction (SAED) accessory, purchased from Thermo Fisher Scientific (Hillsboro, OR, USA). Before analysis, a small amount from each powdery sample was dispersed in deionized water under ultrasonic treatment for 10 min, then 10 µL of solution was placed onto a 400-mesh lacey carbon-coated copper grid and left to dry at room temperature. The microscope was operated at a voltage of 200 kV.

#### 4.3.4. Dynamic Light Scattering (DLS)

For hydrodynamic diameter and zeta potential measurements, a DelsaMax Pro device from Backman Coulter (Brea, CA, USA), equipped with a 532 nm laser, was used. For sample preparation, the powder samples were dispersed in ultrapure water under sonication (10 min), and then as-obtained suspensions were injected into the equipment measurement cell. Six individual acquisitions were recorded for each measurement.

#### 4.3.5. Fourier Transform Infrared Spectroscopy (FTIR)

For compositional investigation, functional groups within the synthesized materials were identified using FTIR analysis. The equipment utilized was a Thermo iN10-MX FTIR spectrometer (Thermo Fisher Scientific, Waltham, MA, USA) coupled with ZnSe crystal. The measurements were performed in the range of 4000–400 cm^−1^ at 4 cm^−1^ resolution, with 32 scans per sample, then processed and converted to absorbance using the OmniPicta 8.2 software (Thermo Fisher Scientific).

### 4.4. Functional Investigation Methods

#### 4.4.1. Ultraviolet–Visible (UV–Vis) Spectroscopy

The ultraviolet–visible (UV–Vis) spectroscopy analysis was used to evaluate the release of oxygen from proposed hydrogels in time. The equipment used in our experiments was an Evolution 300 double-beam spectrophotometer from Thermo Fisher Scientific (Waltham, MA, USA), and data processing was performed with the included 4.5.0 VisionPro software.

#### 4.4.2. Oxygen Bubble Visualization

Two phosphate buffer saline (PBS) solutions with different pH (6 and 7.4) were used to observe the formation of oxygen bubbles. Volumes of 1.5 mL from each PBS solution were placed in Eppendorf tubes, and 500 µL of CaO_2_@TA dispersed in ethanol were added to each tube. For hydrogel formulations, Eppendorf tubes containing 1 mL of each PBS solution were used, and then a volume of 10 µL of hydrogel was added. A final observation on the oxygenation within hydrogel samples (Alg, Alg_S(15_15), Alg_CaO_2_@TA, and Alg_S_O) was recorded after 7 days of testing at room temperature. The visualization of oxygen bubbles was possible using the 12 MP camera of a Galaxy Note9 smartphone (Samsung Electronics, Suwon, Republic of Korea).

#### 4.4.3. Swelling Rate

To evaluate the degree of swelling, lyophilized cross-linked hydrogel samples with similar shapes and sizes were prepared using a punch with 5 mm diameter. The evaluation was carried out with the aim to simulate the behavior of the proposed materials when in contact with body conditions. In this respect, samples were tested at 37 °C after incubation in simulated body fluid (SBF), which was freshly prepared by handling the Kokubo protocol. Formula (2) was used to determine the capacity of hydrogels to swell by considering the initial dry mass of samples (Wi) and the final wet mass of samples after SBF incubation for different times (Wt).

(2) $$Swelling\;rate\;(\%)=\frac{Wt-Wi}{Wi}\times 100\%$$

#### 4.4.4. Degradation Rate

This evaluation, representing the starting step of degradation, was assessed by taking into consideration the first decrease in mass in swelling measurements. This hydrogel parameter was estimated by the initial dry mass of samples (W0) and the mass of samples after swelling and subsequent drying at 60 °C (Wt), according to Formula (3).

(3) $$Degradation\;(\%)=(1-\frac{W0-Wt}{W0})\times 100\%$$

### 4.5. Microbiological Assessment

The effects of the proposed materials on the development of biofilms were evaluated at different times of exposure against two bacterial species that are relevant for the generally known wound infection, namely Gram-positive *Staphylococcus aureus* ATCC 25923 and Gram-negative *Pseudomonas aeruginosa* ATCC 27853. The availability of bacterial strains was from the culture store of the Microbiology Immunology Department of the Faculty of Biology, University of Bucharest, as glycerol stocks at −80 °C. The lyophilized samples (5 mm diameter disks) were firstly exposed to ultraviolet light for 20 min (for sterilization), then individually distributed in each well of 24 multi-well plates (Nunc, St. Louis, MO, USA). Each well was filled with 2 mL of nutritive broth, and microbial suspensions of 10^5^–10^6^ colony-forming units (CFU/mL) were inoculated. In order to assess the time-dependent dynamic development of biofilms in contact with the proposed materials, 24 and 48 h were the intervals considered for incubation at 37 °C. After incubation, the materials were meticulously washed with sterile saline buffer to discard unattached microbial cells. The next step was the biofilm detachment, attained by immersing the materials in 1.5 mL Eppendorf tubes containing sterile saline buffer, followed by vigorous vortexing and additional sonication (30 and 10 s, respectively). For the resulting biofilm, detached cell suspensions were used to perform serial 10-fold dilutions, then 10 µL of each final dilution was inoculated on nutritive agar to count the viable cells, and the colony-forming unit (CFU/mL) values for each sample were acquired. Antimicrobial results were processed with the GraphPad Prism software (GraphPad v9), using the one-way ANOVA statistical tool. Statistical analysis was conducted on three distinct sample sets.

### 4.6. Biological Evaluation

The biocompatibility of the novel synthesized materials was investigated using the HaCaT non-tumor keratinocytes cell line. Cells were cultured in Dulbecco’s Modified Eagle Medium (DMEM, Sigma-Aldrich), supplemented with 10% fetal bovine serum (FBS, Gibco) and 1% penicillin–streptomycin antibiotic mixture (Sigma-Aldrich), and maintained in standard cell culture conditions (37 °C, 95% humidity, and 5% CO_2_) throughout the experiments. The hydrogels were used in the form of a film of approximately 2 mm deposited on 48-well plates. Before cell seeding, samples underwent sterilization through 20 min of UV exposure. Subsequently, cells were seeded onto the materials at an initial density of 2.5 × 10^4^ cells/cm^2^ and further incubated for 72 h in standard cell culture conditions. The experimental control was represented by the Alg sample for all biological investigations.

To evaluate the viability and proliferation of human keratinocytes after 24 h and 72 h of contact with the novel materials, the MTT assay was employed. Briefly, the cell culture medium was replaced with a serum-free DMEM solution containing MTT ([3-(4,5-dimethylthiazol-2-yl)-2,5-diphenyltetrazolium]) (Sigma-Aldrich) at a final concentration of 1 mg/mL. Samples were then incubated for 4 h at 37 °C, 95% humidity, and 5% CO_2_. The resulting formazan crystals were dissolved in isopropanol, and the absorbance of the as-obtained solutions was measured at 550 nm using the FlexStation III multimodal plate reader (Molecular Devices, San Jose, CA, USA). Results are expressed as % cell viability relative to the mean optical density (OD) of the experimental control 24 h post-seeding, which was considered 100% cell viability.

To assess the cytotoxic potential of the hydrogel formulations, the culture medium was harvested after 24 h and 72 h of incubation and processed using the TOX-7 kit (Sigma-Aldrich) to reveal lactate dehydrogenase (LDH) levels released by the cells due to potential cellular membrane damage. The culture supernatants were mixed with the kit reactants according to the manufacturer’s instructions, then incubated for 30 min in the dark. After the reaction was stopped by adding 1/10 volume of 1N HCl to each well, the solutions’ absorbance was measured at 490 nm using the FlexStation III multimodal plate reader (Molecular Devices). Results are presented as % LDH release relative to the mean OD of the experimental control 24 h post-seeding, which was considered 100% cytotoxicity.

Nitric oxide (NO) levels in the culture medium were measured using the Griess Reagent System (Promega) after 24 and 72 h of cell-material incubation. The assay is based on measuring nitrite levels (NO_2_^−^), a primary, stable, and nonvolatile breakdown product of NO. Briefly, culture supernatants were mixed with sulfanilamide and NED solutions, incubated according to the manufacturer’s instructions, and then the absorbance was measured at 550 nm using the FlexStation III reader. The nitrite standard reference curve was prepared using the 0.1 M nitrite standard, as recommended by the manufacturer’s instructions, to calculate NO_2_^−^ concentration in cell culture medium samples.

The morphology of HaCaT cells following 24 and 72 h of incubation with the experimental samples was examined using fluorescence microscopy after staining with AlexaFluor 594-phalloidin (Sigma Aldrich) and 4,6-diamidino-2-phenylindole (DAPI). Initially, samples were treated in a 4% paraformaldehyde solution (PFA, Sigma Aldrich) for 20 min to ensure cell fixation. Subsequently, they were treated with a 2% BSA solution containing 0.1% Triton ×100 for permeabilization. After permeabilization, samples were stained with AF594-conjugated phalloidin for 1 h at 37 °C for F-actin filaments staining, followed by a 20-min incubation time with DAPI for nuclear staining. Imaging was conducted using the Olympus IX73 fluorescent microscope (Olympus Life Science, Waltham, MA, USA), and images were captured and processed using CellSense F software (version 1.11).

## 5. Conclusions

The aim of this study was to develop a suitable hydrogel dressing for the treatment of chronic wounds, by considering the well-known antimicrobial role of silver (S) nanoparticles along with tannylated calcium peroxide (CaO_2_@TA) nanoparticles for oxygen release, by embedding them in a biocompatible alginate (Alg) matrix. The physicochemical investigations revealed the spherical morphology and dimensional uniformity of both types of nanoparticles (SEM and TEM results), in which the sole crystalline phase consisted of cubic silver (S(15_15)) and tetragonal calcium peroxide (CaO_2_@TA), as complementarily evidenced by XRD and SAED data. DLS measurements exposed the negative charge of S(15_15) silver (−7.62 mV) and CaO_2_@TA nanoparticles (−16.48 mV), which is proper for beneficial cell interactions, thus highlighting their stability. Moreover, the tannylated nanoparticles determined an accelerated oxygen bubble formation under acidic conditions, exhibiting a time-dependent and pH-mediated oxygenation ability. The alginates-based hydrogels showed a porous structure with micron-sized porosity, along with a uniform distribution of silver and tannylated calcium peroxide nanoparticles within the polymer matrix. The swelling rate evaluation indicated a proper capacity of the proposed hydrogel formulations to absorb quite a large amount of SBF, and the UV–Vis analysis confirmed the oxygen-releasing ability of Alg embedding S(15_15)) and CaO_2_@TA (Alg_S_O), which recorded a maximal gradual release of about 5.5 mg/L (10.8 µM) H_2_O_2_ during eight hours of measurements. The biofilm modulation assay indicated a synergistic effect between silver and tannylated calcium peroxide nanoparticles, having an impressive antibiofilm efficiency against the *S. aureus* bacterial strain after 48 h of incubation. The biological evaluation data evidenced the favorable behavior of healthy keratinocytes in contact with the obtained materials, with superior characteristics demonstrated for the Alg_S_O sample. Overall, our study adopted advanced synthesis methods for developing smart wound dressings and demonstrated their efficiency in impairing opportunistic infection susceptibility and improving oxygenation-mediated cellular events, thus validating them for promising use in the management of chronic wounds.

## Figures and Tables

**Table 1:** Crystallite size results for S(15_15), S(15_30), S(30_15), and S(10_15) samples.

Sample	S(15_15)	S(15_30)	S(30_15)	S(10_15)
Average crystallite size (nm)	21.27	21.27	20.47	21.69

**Table 2:** Sample coding for silver samples depends on the flowing parameters of reactant solutions within the microfluidic platform.

AgNO_3__PVP Flow Channel	C_6_H_8_O_6_ Flow Channel (×2)	Sample Code
15 RPM	15 RPM	S(15_15)
15 RPM	30 RPM	S(15_30)
30 RPM	15 RPM	S(30_15)
10 RPM	15 RPM	S(10_15)

## Data Availability

Data are contained within the article.
